# *Vermilion* and *cinnabar* are involved in ommochrome pigment biosynthesis in eyes but not wings of *Bicyclus anynana* butterflies

**DOI:** 10.1038/s41598-023-36491-9

**Published:** 2023-06-09

**Authors:** Shaun Hong Chuen How, Tirtha Das Banerjee, Antόnia Monteiro

**Affiliations:** Department of Biological Sciences, National University of Singapore, Singapore, 117557 Singapore

**Keywords:** Developmental biology, Evolution, Molecular biology

## Abstract

If the same pigment is found in different tissues in a body, it is natural to assume that the same metabolic pathways are deployed similarly in each tissue. Here we show that this is not the case for ommochromes, the red and orange pigments found in the eyes and wings of butterflies. We tested the expression and function of *vermilion* and *cinnabar*, two known fly genes in the ommochrome pathway, in the development of pigments in the eyes and in the wings of *Bicyclus anynana* butterflies, both traits having reddish/orange pigments. By using fluorescent in-situ hybridization (HCR3.0) we localized the expression of *vermilion* and *cinnabar* in the cytoplasm of pigment cells in the ommatidia but observed no clear expression for either gene on larval and pupal wings. We then disrupted the function of both genes, using CRISPR-Cas9, which resulted in the loss of pigment in the eyes but not in the wings. Using thin-layer chromatography and UV–vis spectroscopy we identified the presence of ommochrome and ommochrome precursors in the orange wing scales and in the hemolymph of pupae. We conclude that the wings either synthesize ommochromes locally, with yet unidentified enzymes or incorporate these pigments synthesized elsewhere from the hemolymph. Different metabolic pathways or transport mechanisms, thus, lead to the presence of ommochromes in the wings and eyes of *B. anynana* butterflies.

## Introduction

Ommochromes are red/orange pigments found in a variety of species that serve diverse functions. They are present in pigment-containing cells of crustaceans, spiders, and insects and play important roles in color patterning, visual filtering, UV protection, and tryptophan detoxification^1^. Ommochromes found in the pigment cells of insect ommatidia, the optical unit of insect eyes, play important roles in the protection of photoreceptor cells and in overall pigmentation of eyes^1–3^. Ommochromes found in the red and orange color patches in the wings of butterflies, such as *Elymnias hypermnestra tinctoria*^4^, *Junonia coenia*^5,6^, and *Agraulis vanilla*^7^ butterflies, likely serve a mate attraction or predator avoidance signalling role.

Ommochromes are produced via the ommochrome biosynthesis pathway, whose enzymes have been mostly studied in the eyes of insect model systems* s*uch as *Drosophila melanogaster*^8^, *Tribolium castaneum*^9^, *Aedes aegypti*^10^, and the lepidopterans *Bombyx mori*^11^ and *Plutella xylostella*^12^. In this pathway, the amino acid tryptophan is converted into the pigments xanthommatin and dihydro-xanthommatin that can be yellow to red depending on the cell’s reducing environment^13^. In *Drosophila*, four key genes coding for different enzymes in this pathway include *vermilion, kynurenine formamidase* (*kfase*), *cinnabar,* and *cardinal*^12^*.* After tryptophan is incorporated into pigment cells by the putative monocarboxylate transporter karmoisin^14^, *vermilion* encodes tryptophan oxygenase which converts tryptophan to formylkynurenine^15^. *kynurenine formamidase* (*kfase*) encodes an eponymous enzyme which converts formylkynurenine to kynurenine^16^. *cinnabar* encodes kynurenine 3-hydroxylase which converts kynurenine to 3-hydroxykynurenine^12^. Finally, *cardinal* encodes phenoxazinone synthetase which catalyses conversion of 3-hydroxykynurenine to xanthommatin (orange), which can be further converted to dihydro-xanthommatin (red), under reductive chemical conditions^13^*.*

Some of the same ommochrome enzyme-coding genes, ommochrome metabolite precursors, and an eye ommochrome transcription factor regulator, have also been identified in the wings of a few nymphalid butterfly species. In *Vanessa cardui*, tryptophan is incorporated in the red and beige colored domains of the wing^17^, and expression of both *vermilion* and *cinnabar* is present in the red color pigmented areas of developing *Heliconius erato* wings^18^. Later studies have identified *optix* as one of the key transcription factors regulating presence of ommochrome pigments in butterflies^19^, because its knockout resulted in the loss of red and orange color pigmentation in multiple species of butterflies, as well as led to the down-regulation of ommochrome pathway-associated genes in wings^20^. The direct role of *vermilion* and *cinnabar* in producing ommochrome pigments in the wings of butterflies, however, has not been tested.

The connection between *optix* and known ommochrome pathway genes is also unclear in *Bicyclus anynana* butterflies, which due to its sequenced genome and ample genetic tools for expression and functional analysis is a good system to study such a pathway. *optix* is clearly involved in the pigmentation of the future orange scale cells in the wing eyespot patterns of this species, as its knockout results in the loss of orange color^21^. But, it is unclear whether the known ommochrome pathway genes are required for orange pigment production in *B. anynana* wings. *vermilion* has been identified in the bulk mRNA extract of *B. anynana* pupal wings at a few time points^22^, but *vermilion* knockouts using CRISPR-Cas9 have not shown any visible effect on the wings^23^. Furthermore, knockout of the ommochrome transporters *white* and *scarlet* did not produce any visible phenotype on the wings either, despite affecting ommatidial pigmentation^23^. These results may indicate (1) that ommochrome pathway genes are expressed in just a small number of cells on the wing of *B. anynana* (the orange ring in eyespots), which so far have not been hit with the CRISPR tool; (2) that ommochromes are not the orange pigments in *B. anynana*; or (3) that ommochromes are not being produced in the wings of *B. anynana,* but are instead being transported there from other ommochrome-producing cells.

To examine the involvement of known ommochrome pigment genes in the wings of *B. anynana*, we examined the expression and function of *vermilion* and *cinnabar* using fluorescent in-situ hybridization and CRISPR-Cas9, respectively. We also examined the expression and function of both genes in the developing eyes, as a control. Our results indicate that these two ommochrome enzymes are essential for eye pigmentation but are not playing any major role in local wing pigment synthesis in *B. anynana*. Next, using thin-layer chromatography and UV–visible spectrometry, we identified the presence of ommochrome pigments and ommochrome precursors in the orange ring of the eyespots and in the hemolymph of *B. anynana,* respectively. We conclude that ommochrome pigments are either being incorporated into the wing from another source, or that novel enzymes are being used for ommochrome synthesis in the wing tissue of this butterfly.

## Results

### Expression of *vermilion* and *cinnabar* in the eyes of *B. anynana*

**Figure 1:**
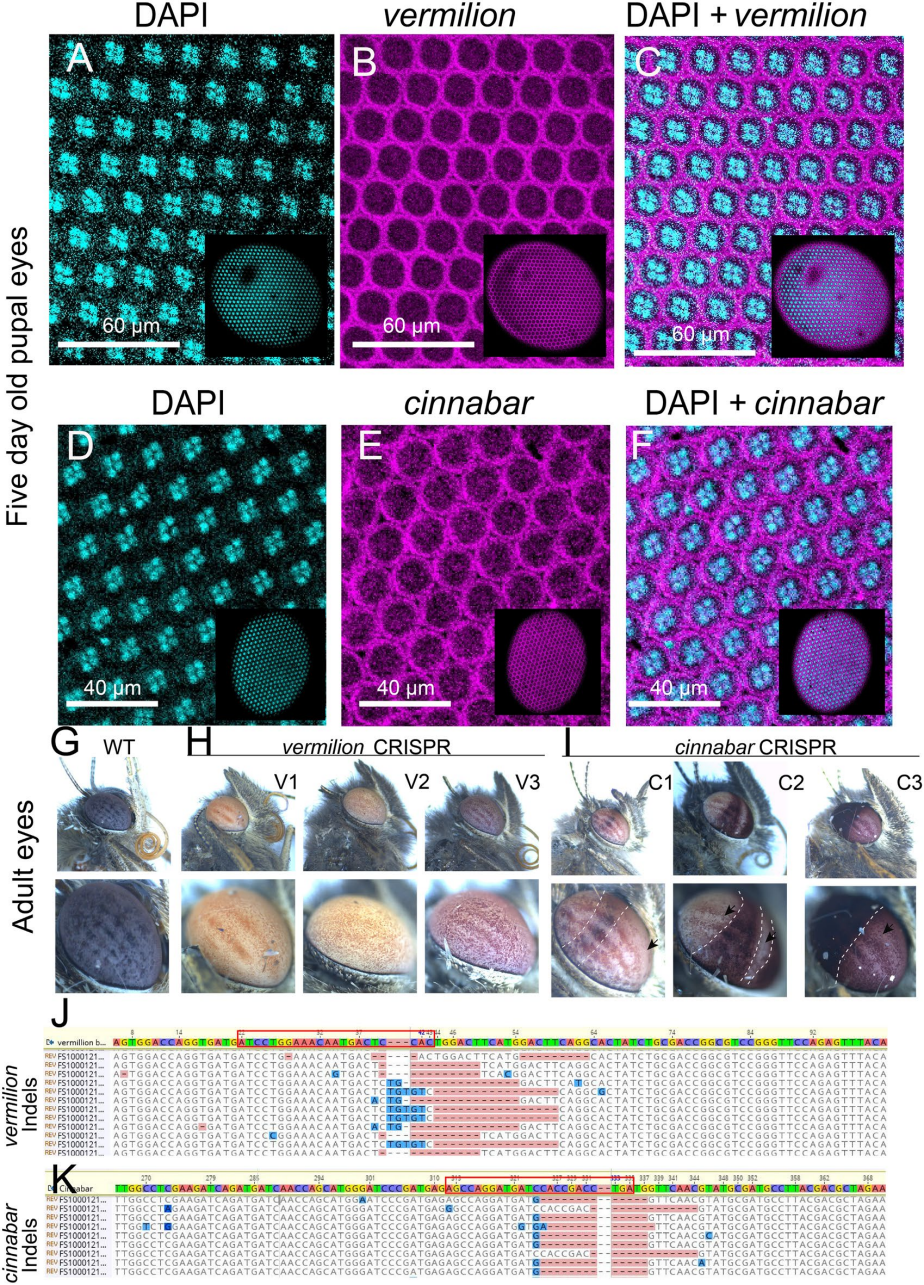
Expression and function of *vermilion* and *cinnabar* in the eyes of *Bicyclus anynana* at 77% pupal development (PD) (120 h). Expression of (**A**) DAPI and (**B**) *vermilion* in the eyes. (**C**) Merged expression of DAPI and *vermilion*. Expression of (**D**) DAPI and (**E**) *cinnabar* in the eyes. (**F**) Merged expression of DAPI and *cinnabar*. DAPI is expressed in four nuclei of the ommatidia and *vermilion* and *cinnabar* mRNA in present in the cytoplasm of those cells. Adult (**G**) WT eyes, (**H**) *vermilion* crispant eyes, and (**I**) *cinnabar* crispant eyes. *vermilion* crispants showed more homogeneous mutant phenotypes, while *cinnabar* crispants showed patches of cells with missing pigmentation (black arrow). (**J**) Indels were found near the site targeted by the guide RNA for *vermilion* (red box) and for (**K**) *cinnabar*.

To identify the spatial localization and expression patterns of *vermilion* and *cinnabar*, we first tested the expression pattern in the developing eyes of butterflies using HCR3.0^24^. We observed clear and distinct expression domains in the cytoplasm of the ommatidial pigment cells for both genes (Figs. 1A–F, S1D,E).

### Expression of *vermilion* and *cinnabar* in the developing wings of *Bicyclus anynana*

**Figure 2:**
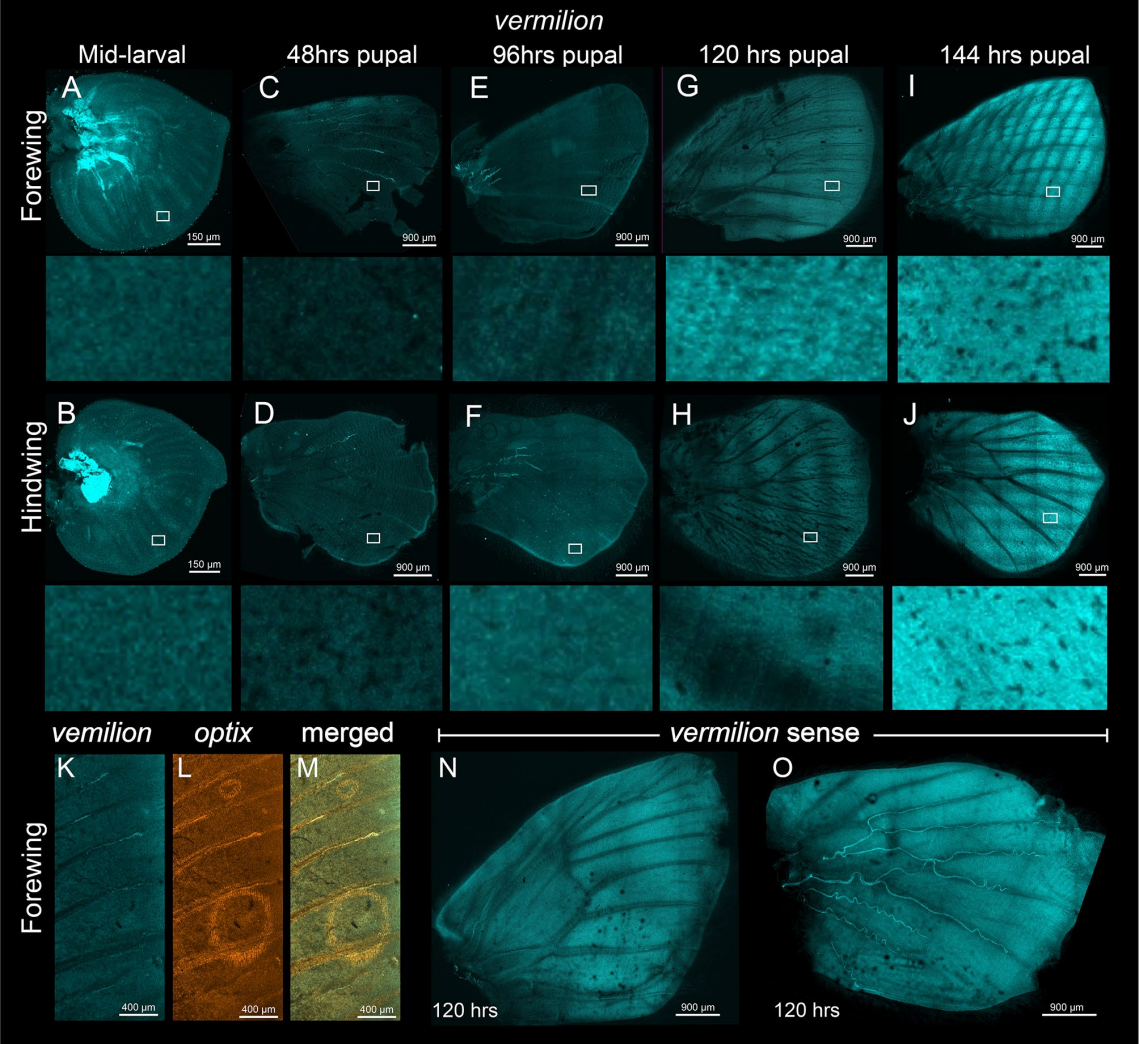
Expression of *vermilion* in the larval and pupal wings of *Bicyclus anynana*. Expression of *vermilion* in the (**A** and **B**) larval wings, (**C** and **D**) 31% pupal development (PD) (48 h), (**E** and **F**) 61.5% PD (96 h), (**G** and **H**) 77% PD (120 h), and (**I** and **J**) 92% PD (144 h). No specific domains of *vermilion* mRNA were observed in the wings. (**K**–**M**) Co-expression of *vermilion* and *optix* (positive control) at 77% PD (120 h). Expression of *vermilion* sense strand in the (**N**) forewing and (**O**) hindwing at 77% PD (120 h).

**Figure 3:**
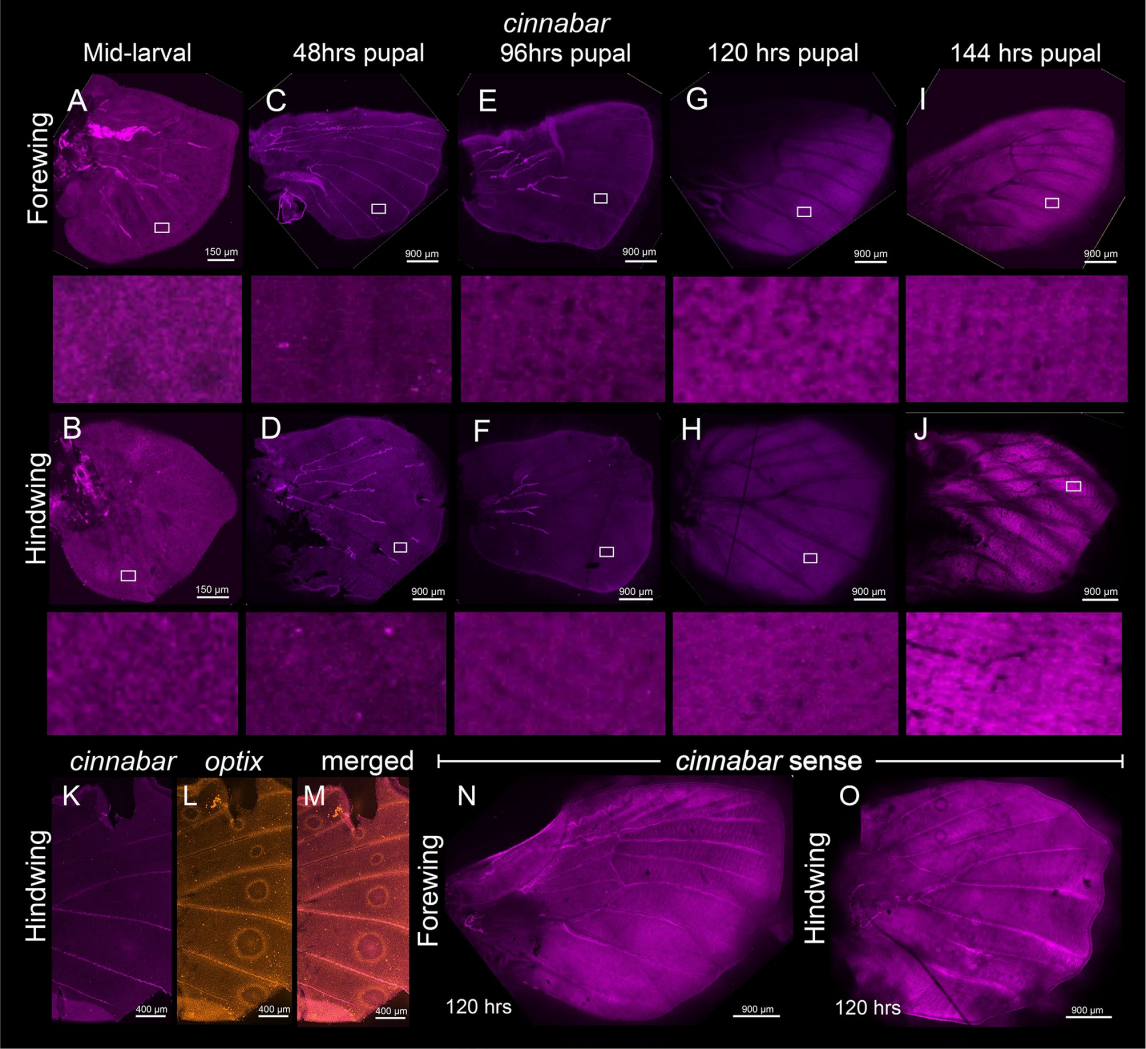
Expression of *cinnabar* in the larval and pupal wings of *B. anynana*. Expression of *cinnabar* in the (**A** and **B**) larval wings, (**C** and **D**) 31% pupal development (PD) (48 h), (**E** and **F**) 61.5% PD (96 h), (**G** and **H**) 77% PD (120 h), and (**I** and **J**) 92% PD (144 h). No specific domains of *cinnabar* mRNA were observed in the wings. (**K**–**M**) Co-expression of *vermilion* and *optix* (positive control) at 77% PD (120 h). Expression of *cinnabar* sense strand in the (**N**) forewing and (**O**) hindwing at 77% PD (120 h).

We then tested the expression of *vermilion* and *cinnabar* in the larval and pupal wings of *B. anynana* using HCR^24^. *vermilion* and *cinnabar* have been previously shown to have higher expression at 40 to 55% pupal development in *Vanessa cardui* and *Heliconius erato*^17,18^, so we examined the expression of these genes up to that stage in *B. anynana*. We were expecting at least a partially overlapping expression domain to that of *optix*, which is expressed in the orange ring of the eyespots during the pupal stage (Figs. 2K–M, 3K–M, S1, S2)^21,25^. None of the genes, however, showed any specific domain of expression in the wings (Figs. 2, 3). At 77% (120 h) and 92% PD (144 h) however, we observed a slight homogeneous higher intensity of fluorescence across the wings, likely due to autofluorescence of chitin during the later developmental stages of pupal wing development, as control stainings showed the same increase in fluorescence (Figs. 2G–J,N,O, 3G–J,N,O).

To verify if the ommochrome genes are transcribed in the 15% PD (24 h) pupal wings, we performed PCR on whole-wing cDNA using primers specific to *vermilion*, *cinnabar* and *kynurenine formamidase*. The data show that mRNAs for these genes are present in pupal wings at low levels (compared to reference *ef1α*) (Fig. S3), consistent with RNA-seq data from a previous study^26^ (Table S5).

### Function of *vermilion* and *cinnabar* in the eyes and wings of *B. anynana*

To validate the function of *vermilion* and *cinnabar* in the eyes and wings of *B. anynana*, we disrupted the coding sequence of both genes using CRISPR. While WT eyes appear as black (Fig. 1G), *vermilion* crispant adults had homogeneous yellow or pink eyes (n = 5) (Fig. 1H), and *cinnabar* crispant adults (n = 6) showed lighter pigmentation in mosaic patches in the eyes (Fig. 1I). Most of these individuals showing eye phenotypes (n = 5 *vermilion,* n = 4 *cinnabar*) (Figs. S4, S15) were confirmed for indels at the CRISPR target site using illumina sequencing of DNA extracted from the eye tissue (Figs. 1J,K, S5–S9, S16–S19).

Crispants of *vermilion* (Figs. 4B–D, S4) and *cinnabar* (Figs. 5B–D, S15) did not produce any wing phenotypes. To confirm if *vermilion* and *cinnabar* were successfully knocked out in the wing, we performed illumina sequencing on the wings of every individual which showed eye pigmentation phenotypes. We obtained indels at the CRISPR target sites in every tested individual (n = 5 *vermilion,* n = 6 *cinnabar*) (Figs. 4E, S10–S14), (Figs. 5E, S20–S25).

**Figure 4:**
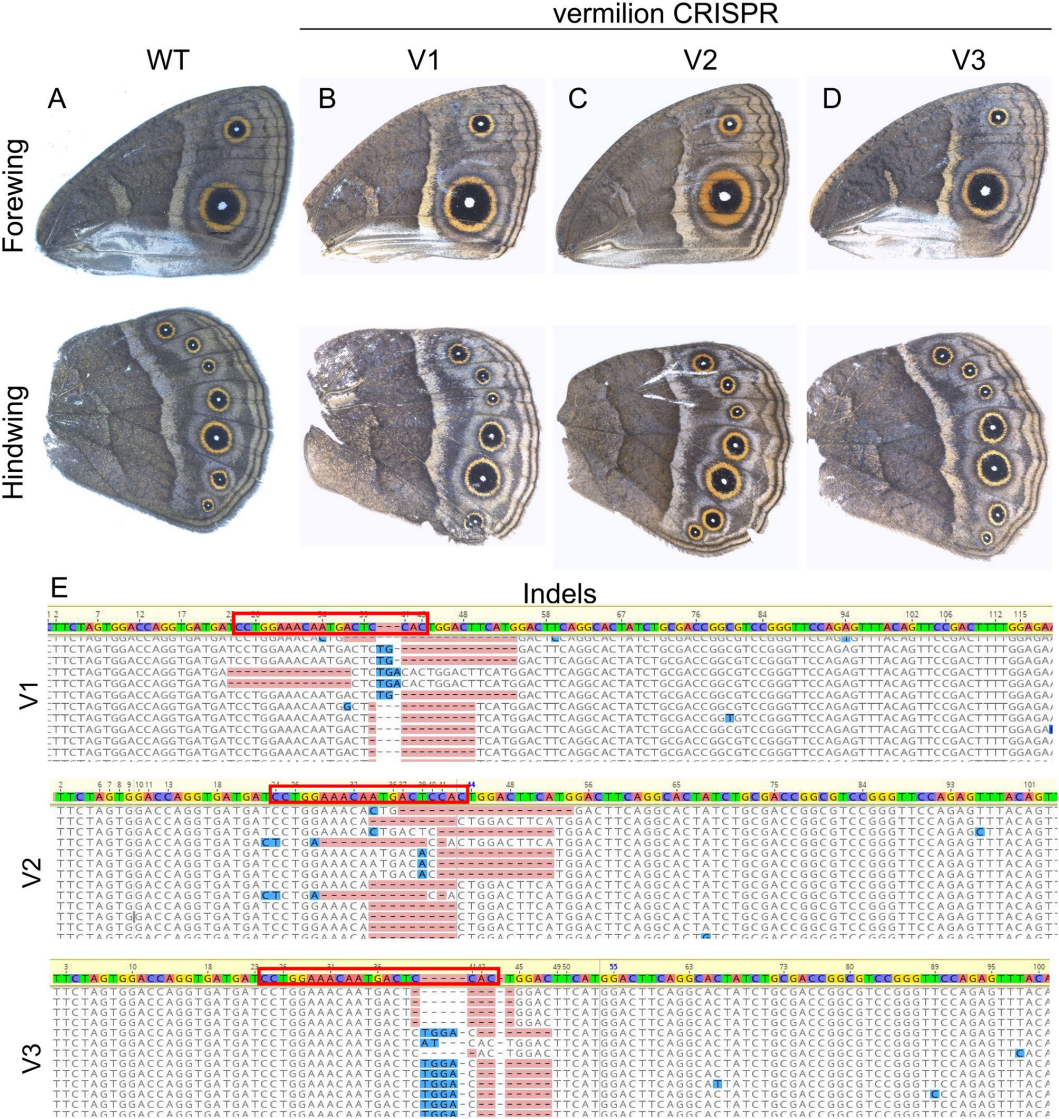
No function of *vermilion* detected in the wings of *Bicyclus anynana*. Adult (**A** and **B**) WT wings, and (**B**–**D**) *vermilion* crispant wings. Indels at the site of (**E**) *vermilion* crispants from the respective wings from (**B**–**D**). No specific phenotypes or mosaic clones were observed in the adult wings. Genetic disruptions at the target site were confirmed via illumina sequencing. Red box highlights the CRISPR target site.

**Figure 5:**
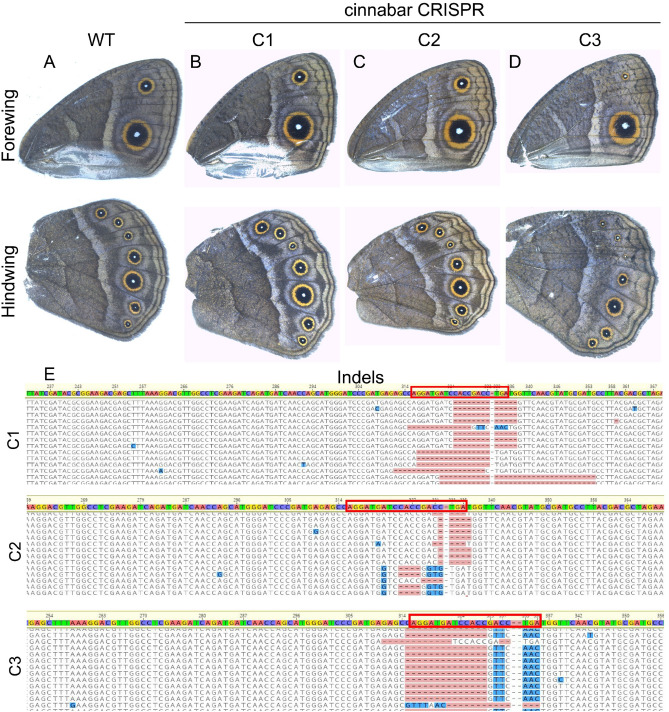
No function of *cinnabar* detected in the wings of *B. anynana*. Adult (**A**) WT wings, and (**B**–**D**) *cinnabar* crispant wings. Indels at the site of (**E**) *cinnabar* crispants from the respective wings from the panel B-D. No specific phenotypes or mosaic clones were observed in the adult wings. Genetic disruptions at the target site were confirmed via illumina sequencing. Red box highlights the CRISPR target site.

### *Kynurenine formamidase *(*kfase*) does not appear to be involved in eye or wing phenotypes

We tested one additional known ommochrome biosynthesis gene, *kynurenine formamidase* (*kfase*), using HCR (Fig. S26) and CRISPR (Fig. S27). In this case, knockouts of the *kfase* did not result in any observable eye or wing phenotypes (n = 5) (Fig. S27), even though the transcripts were clearly localized in the ommatidial cells during pupal development (Fig. S27; Table S5). No known *kfase* mutant has been reported for insect species^1^.

### Ommochromes are likely present in *B. anynana*, *J. almana*, and *J. orythia*

**Figure 6:**
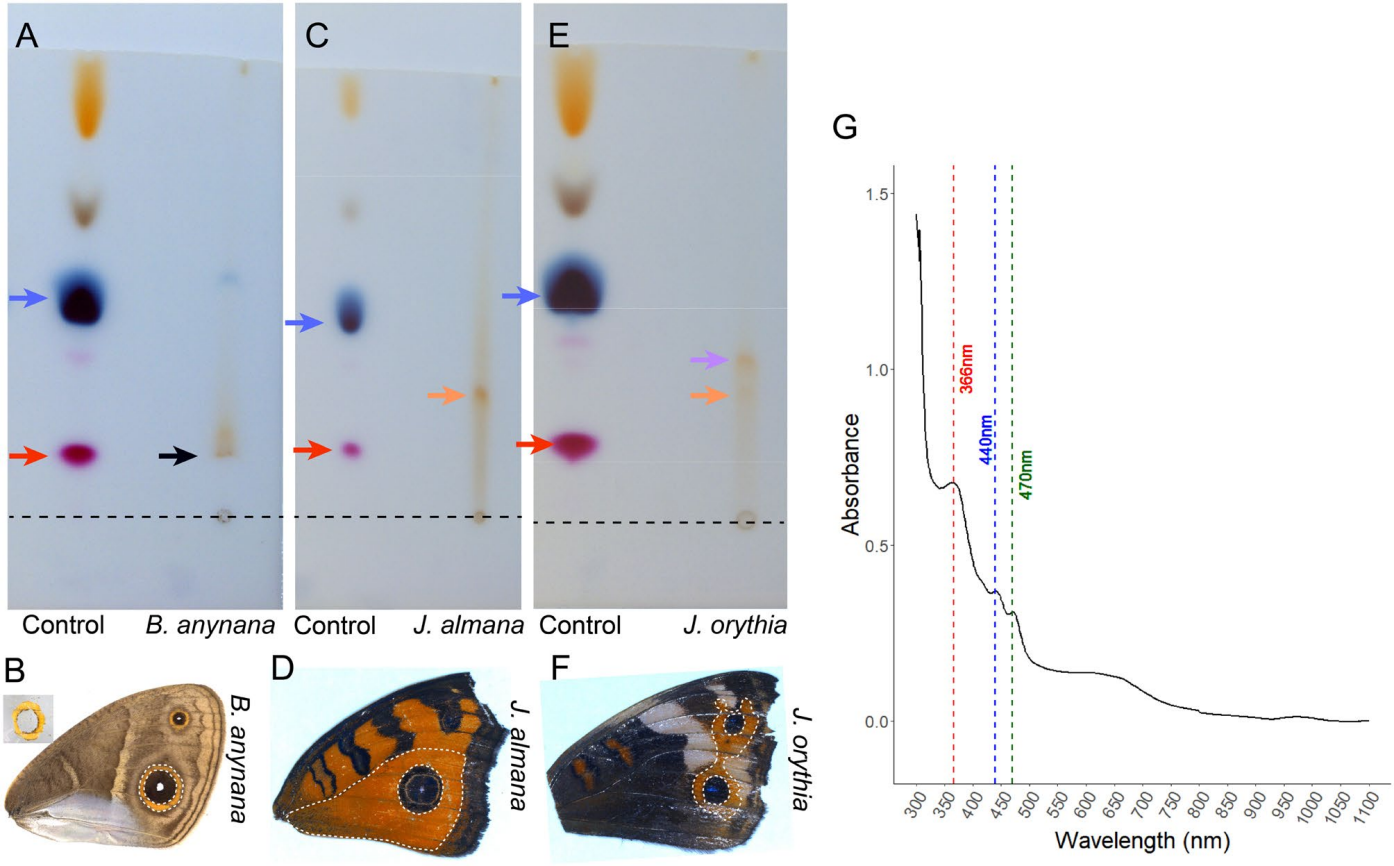
Thin layer chromatography (TLC) on the pigments extracted from the orange sections of *B. anynana*, *J. almana*, and *J. orythia*. (**A**,**B**) Pigments extracted from the orange ring of *B. anynana* showing a band at the retention factor (Rf) value of dihydro-xanthommatin (black arrow). (**C**,**D**) Pigments extracted from the orange ring of *J. almana* showing a band at the Rf value of ommatin-D (orange arrow). (**E**,**F**) Pigments extracted from the orange ring of *J. orythia* showing a band at the Rf value of xanthommatin and ommatin-D (purple and orange arrow). Control pigments amaranth and bromophenol blue migrate as red and blue pigments, respectively (red and blue arrow). Bromophenol blue has two additional brown and yellow bands and amaranth has an additional red band. (**G**) UV–Visible Spectra of 77% (120 h) PD pupal hemolymph pigment extract, showing three peaks marked by colored dashed lines at wavelengths likely corresponding to kynurenine (red, 366 nm), xanthommatin (blue, 440 nm) and dihydro-xanthommatin (green, 470 nm).

Since we could not identify any defects in wing pigmentation with *vermilion* and *cinnabar*, we were curious to test whether ommochrome pigments were in fact present in the orange scale region of adult *B. anynana* wings. We extracted pigments from the orange scales of *B. anynana*, along with pigments from the orange region of two *Junonia* species, as studies on *J. coenia* have previously confirmed ommochromes in their wings^5^. We ran a thin layer chromatography (TLC) experiment with two control pigments with known retention factors (Rf), amaranth and bromophenol blue, to help identify the presence of ommochromes (also with known Rfs)^17^ (Fig. 6). We observed the presence of a pigment with the same chromatography Rf value of dihydro-xanthommatin in *B. anynana* (black arrow, Fig. 6A; Table 1). In *J. almana*, a pigment with the retention properties of ommatin-D was present at higher levels (orange arrow, Fig. 6C; Table 1), while *J. orythia* indicated the likely presence of ommatin-D and xanthommatin (orange and purple arrows, Fig. 6E; Table 1). These results show the presence of ommochromes in *B. anynana* wings despite the absence of function of known ommochrome biosynthetic enzymes in the wing tissue of this species. The pigments in the TLC bands, however, need further validation with mass spectrometry experiments.

**Table 1:** Retention factor (Rf) values of the pigments extracted from *Bicyclus anynana*, *Junonia almana*, and *Junonia orythia*.

Species/plate	Compound	Rf	Theoretical Rf^5^
*B. anynana*	Bromophenol blue	0.43	0.43
*B. anynana*	Amaranth	0.13	0.13
*B. anynana*	Dihydro-xanthommatin	0.13	0.13
*J. almana*	Bromophenol blue	0.44	0.43
*J. almana*	Amaranth	0.14	0.13
*J. almana*	Ommatin-D	0.28	0.29
*J. orythia*	Bromophenol blue	0.44	0.43
*J. orythia*	Amaranth	0.14	0.13
*J. orythia*	Xanthommatin	0.34	0.36
*J. orythia*	Ommatin-D	0.27	0.29

For detailed calculations and raw values see Table S1.

### Ommochromes and kynurenine are found in *B. anynana* pupal hemolymph

To further explore how ommochromes are present in wings of *B. anynana* we tested the hypothesis that ommochromes and their precursors might be taken up from the hemolymph and incorporated into the wing scales during pupal development. We purified the pigments from the hemolymph of individuals at 77% (120 h) PD and obtained their absorbance spectra (Fig. 6G). We observed absorbance peaks at various wavelengths of 366 nm, 440 nm, and 470 nm. Based on previously reported values^27–29^, these wavelengths indicate the likely presence of, kynurenine, xanthommatin, and dihydro-xanthommatin respectively, in the hemolymph. These results indicate that one of the mechanisms whereby ommochromes appear in *B. anynana* wings might be via transport from the hemolymph.

## Discussion

### Ommochrome enzymes control eye pigmentation in *B. anynana*

In *B. anynana*, the ommochrome enzymes *vermilion* and *cinnabar* are expressed in the pigment cells of the ommatidia during pupal development where they function in pigment synthesis. Both genes are transcribed at around 77% PD (120 h) in the eyes (Fig. 1A–F) with transcripts detected as early as 31% PD (48 h) and 46% PD (72 h) (Fig. S1D,E). In each ommatidium, the expression was limited to the periphery, where primary and secondary pigment cells are situated^1^ (Fig. 1A–F). Most *vermilion* crispants had a homogeneous pale orange eye color (Fig. 1H), while *cinnabar* crispants mostly had lighter red patches in their eyes (Fig. 1I). These results are similar to expression studies in other insect species such as *Acheta domesticus* and *Henosepilachna vigintioctopunctata*^30,31^, and knockout studies of *vermilion* or *cinnabar* in *Tribolium castaneum*, *Aedes aegypti*, *Plutella xylostella*, *Nasonia vitripennis*, and *Helicoverpa zea*, which all altered adult ommatidia coloration^9,10,12,15,32^.

### *vermilion* and *cinnabar* do not control pigmentation in the wings of *B. anynana*

Neither *vermilion* nor *cinnabar* are playing a functional role in wing ommochrome synthesis in *B. anynana*, despite the presence of mRNAs encoding for both the enzymes in pupal wings, and the likely presence of ommochromes on the adult wing. In our experiments, no noticeable expression of these genes was apparent in larval and pupal wings and eyespots prior to 92% PD (144 h) (Figs. 2, 3). Functional validation of these two ommochrome enzymes using CRISPR-Cas9 also did not result in any observable phenotype in the wings of *B. anynana* (Figs. 4, 5, S4, S15), despite the presence of a large percentage of reads with indels suggesting successful knockouts in the majority of wing cells (Figs. 4, 5, S10–S14, S20–S25). Both the lack of strong expression of *vermilion* and *cinnabar* across the wing and the lack of CRISPR phenotypes, despite confirmation of genetic disruptions to these genes in every wing of successful eye crispants, suggests that these genes do not play a role in ommochrome synthesis in the wings of *B. anynana*. Whether or not these genes play a role in local ommochrome synthesis in the wings of other species, such as *Heliconius erato*, *Vanessa cardui*, and *Junonia coenia*^17,18,20^ also awaits direct functional tests with both genes.

The presence of functionally redundant ommochrome synthesis genes will need to be explored further in *B. anynana*. Such functional redundancy might explain the lack of phenotypes in wings, where the role of *vermilion* and *cinnabar* might be taken up by other genes. The lack of clear expression of *kfase* in WT wings, and of mutant phenotypes for *kfase* crispants, suggests that this gene might also not be functioning in ommochrome synthesis on the wings of *B. anynana*. In these crispants, however, we did not confirm the presence of indels. In addition, the function of other pathway enzymes such as *cardinal* has yet to be investigated in *B. anynana*.

### Alternative pathways of ommochrome pigmentation biosynthesis or transport in *B. anynana*

Here we have shown that ommochrome pigments are likely a part of the orange ring in the wings of *B. anynana*. Ommochromes were proposed to be involved in coloring the orange ring of the eyespots in this species based solely on the expression and functional data of *optix*^21^, an upstream regulator of ommochromes in other butterflies^20^. Using thin layer chromatography, however, we have identified a small amount of a pigment, in the orange scale region of the eyespots, that matches (in Rf value) the ommochrome dihydro-xanthommatin. The identity of these ommochromes is also probably distinct from ommochrome pigments present in the wings of *Junonia*. These ommochromes, however, do not appear to be deposited in wing scales in visible pigment granules as observed via SEM^33^ (Fig. S28), as described for the ommatidial pigment cells in insect eyes^34^. The precise molecular identity of the pigments observed using TLC, however, will need further confirmation using mass-spectrometry.

**Figure 7:**
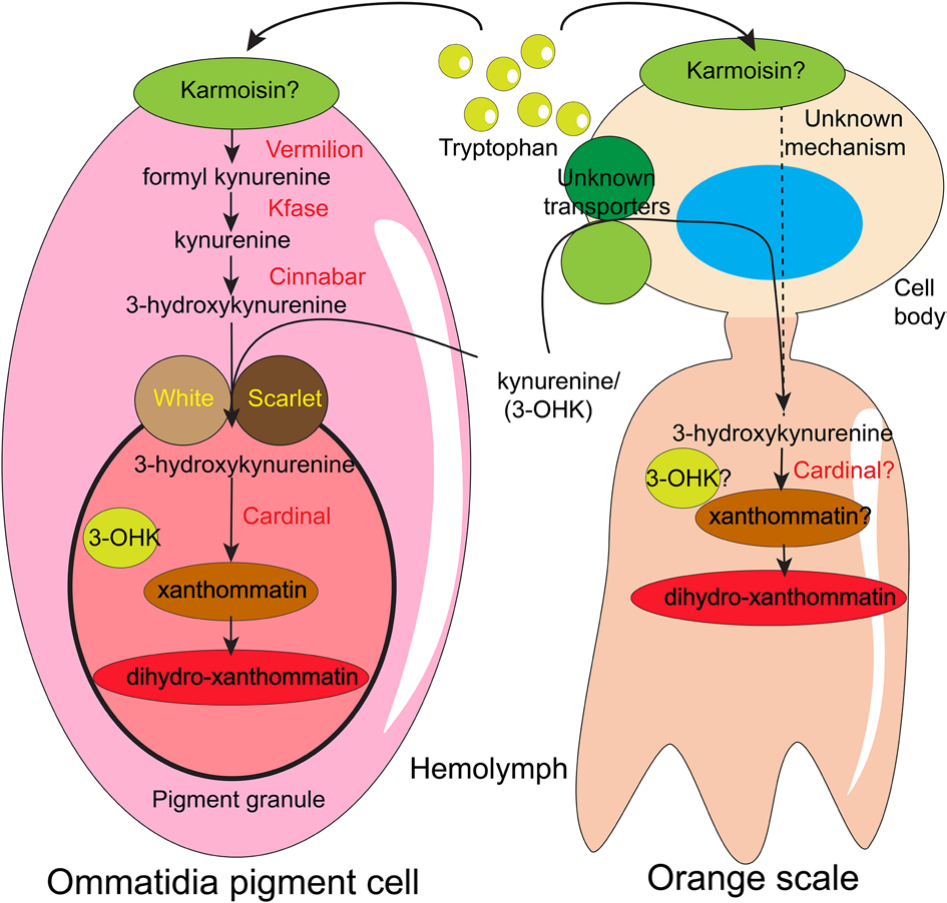
Mechanism of ommochrome biosynthesis and/or uptake in the ommatidia pigment cells and orange scale cells of *Bicyclus anynana* butterflies. In the ommatidia pigment cells tryptophan is taken up by the proposed karmoisin transporter where in the presence of Vermilion*,* Kfase*,* and Cinnabar enzymes*,* it is converted to 3-hydroxykynurenine (3-OHK). 3-OHK is taken up inside the pigment granule by transporter proteins White and Scarlet where it is converted to xanthommatin and dihydro-xanthommatin. In the orange scales either tryptophan is converted to 3-OHK, by an unknown mechanism, or is transported from the hemolymph via unknown transporters to the scale cells where it is converted to xanthommatin and dihydro-xanthommatin. We also propose the absence of pigment granules for ommochromes in scale cells. Note there is no direct evidence regarding the involvement of karmoisin in tryptophan uptake in *Drosophila.*

In *B. anynana*, the ommochrome and/or ommochrome precursors found on the wings may be synthesized in another organ, transported via the hemolymph, and be taken up via ommochrome transporters into the orange scales (Fig. 7). A similar transport mechanism has been hypothesised for the precursor 3-hydroxykynurine in the wings of *Heliconius* butterflies^18^. Early studies have demonstrated that metabolites of ommochrome biosynthesis can be secreted from one tissue into the hemolymph and processed by another tissue type. In *Ephestia kühniella* larvae, *kynurenine 3-hydroxylase* (*cinnabar*) activity was solely localised in the Malpighian tubules while its enzymatic product 3-hydroxykynurenine was detected in the larval haemolymph, together with earlier metabolites in the pigment pathway, such as tryptophan and kynurenine^35^ (Fig. 7). Pupal ommatidia of this species are capable of taking up hemolymph-borne 3-hydroxykynurenine to produce ommochromes when the metabolite is injected into the hemolymph^36^. In *Araschnia levana* butterfly pupae, an increase in 3-hydroxykynurenine was found in the haemolymph as red pigments appeared in the wing scales, and injection of radiolabelled 3-hydroxykynurenine revealed that its incorporation into the wing coincided with the spatial localization of red scales^37^. In *Papilio xuthus*, kynurenine circulates freely in the hemolymph during pupal development and increases in concentration as ommatidial ommochrome formation initiates, and decreases sharply as a red wing color appears^38^. These studies suggest that ommochromes found in the wings (and eyes) of these divergent moth and butterfly species may derive from the hemolymph.

An alternative mechanism to explain the presence of ommochromes in wings that lack the expression of *vermilion* and *cinnabar*, could involve the use of distinct enzymes in wings and eyes (Fig. 7). In *Vanessa cardui*, differential gene expression analyses performed across different colored regions of the wing identified 26 genes potentially involved in ommochrome pigmentation. These genes include *optix*, *kfase*, *cinnabar*, seven major facilitator superfamily (MFS) transporters, two juvenile hormone-binding proteins and two unclassified transporters^39^. Notably, ommochrome transporter genes *white* and *scarlet* were not found to be differentially expressed between the tested timepoints and the differently colored areas of *V. cardui* wings. In *Heliconius*, the expression of three novel transporter genes was also found associated with red wing patterns^40^. In *B. anynana*, CRISPR knockouts of ommochrome transporters *white* and *scarlet* did not lead to any visible phenotype on the wings either^23^, hinting at the possibility of novel enzymes as well as ommochrome transporters present in the scale cells of all these species. Thus, it is possible that over the course of evolution distinct enzymes and transporter proteins, have been deployed in *B. anynana* wings, relative to those used in the eyes.

In conclusion, we have shown the involvement of the ommochrome biosynthesis enzymes *vermilion* and *cinnabar* in the local production of pigments in the eyes but not in the wings of *Bicyclus anynana* butterflies. These enzymes might still be involved in the production of the ommochrome pigments that are eventually deposited in the orange areas of the wings, but they don’t appear to be functional in the wing cells themselves. Ommochrome pigments or pigment precursors are either transported into the scale cells, after the two enzymatic steps investigated, or are synthesized in situ via novel enzymes.

## Methods

### Hybridisation chain reaction (HCR3.0) of wings and eyes

HCR3.0 was carried out based on previously described protocols^24^. Briefly wing and compound eye tissues at the desired post-pupation timepoints were dissected in 1X PBS solution at room temperature and fixed in glass wells containing 4% Formaldehyde in 1X PBS. After 30 to 40 min of fixation at room temperature, tissues were washed twice in 1X PBS for 3 min and then twice in 1X PBS supplemented with 0.1% Tween 20 (1X PBST). Permeabilization was performed by incubating tissues for 30 min in a detergent solution containing 1.0% SDS, 0.5% Tween 20, Tris–HCl (pH 7.5), 1.0 mM EDTA (pH 8.0) and 150.0 mM NaCl. Late-stage (> 60% pupal development) pupal wings with thicker cuticles were digested in 2.5 μL Proteinase K in 200 μL 1X PBST for 2 min at 55 °C in order to enhance tissue permeability for probe entry. Subsequently, the wings were placed on ice and the digestion mix was replaced with 2 mg/mL glycine in 1X PBST was added to stop the reaction. Tissues were then washed thrice with 1X PBST and twice with 5X SSCT. The tissues were incubated in 30% probe hybridization buffer at 37 °C for 30 min, before a longer incubation in 30% probe hybridization buffer with 0.02 μM primary probes (specific to *vermilion, cinnabar*, and *kfase*) at 37 °C for 16 h. Tissues were washed four times in 30% probe wash buffer in 15-min intervals at 37 °C and washed twice with 5X SSCT at room temperature. The tissues were incubated in amplification buffer for 30 min at room temperature and subsequently in amplification buffer with secondary fluorescent probes in the dark at room temperature for 12 h. The tissues were then washed in 5X SSCT for four times in 20-min intervals, incubated with DAPI diluted in 5X SSCT for 5 min and washed twice with 5X SSCT. The tissues were mounted on a glass slide in mounting buffer and imaged with an Olympus FV3000 confocal microscope.

### CRISPR-Cas9

CRISPR-Cas9 gene editing for *vermilion, cinnabar* and *kynurenine formamidase* were performed in line with previously described protocol^41^ with the following minor adjustments. For all the genes*,* synthetic guide RNAs (crRNA) were designed (sequences in Supplementary File) using the IDT custom guide design tool. A solution comprising duplex buffer, Cas9 Buffer and equimolar mixtures of crRNA and Alt-R® CRISPR-Cas9 tracrRNA were incubated at 95 °C for 5 min and cooled to room temperature prior to addition of Cas9 nuclease.

### Detection of indels

We confirmed the presence of *vermilion* and *cinnabar* mutations both in pupal and adult tissues. Pupal wing tissues were dissected from individuals either at 25 to 28% (40–44 h) or 44 to 47% (70–74 h) pupal development. DNA was extracted and purified using the Omega Bio-tek E.Z.N.A Tissue DNA Kit (SKU: D3396-01). In adult crispants, eye and wing tissues were isolated and both tissues types were processed separately using the DNA extraction techniques as mentioned above. Paired-end sequencing was performed on sequencing libraries comprising of the amplified region of interest and the presence of INDELs were verified with Geneious v10.1.3.

### Thin layer chromatography

Pigment extraction from wing sections was performed following a previously defined protocol^5,17^. The orange scale regions from the wings were isolated from the adult wing of *B. anynana*, *J. almana* and *J. orythia* using fine scissors (Fig. 6). Dissected wing tissues were homogenised in acidified methanol (0.5% HCl) using 0.01 mm zirconium beads in a homogenizer (Next Advance Bullet Blender). The homogenate was centrifuged at 14,000 rpm for 5 min and the supernatant was desiccated in a vacuum centrifuge (ThermoScientific) for 30 min and reconstituted in 20 µl methanol. The pigment mixtures, as well as reference dyes amaranth and bromophenol blue, were spotted on a silica gel plate (F254, Merck) and ran with phenol (SigmaAldrich) as the developing solvent. Retention factor (Rf) values were calculated as the ratio of the migration distance of the pigment compound to that of the solvent front measured on ImageJ.

### UV–visible spectroscopy

Upon rearing to 61.5% pupal development, pupal haemolymph was extracted from wild-type *B. anynana* individuals. Ommochrome pigments were extracted from pupal hemolymph using cold methanol following a previously defined protocol^18^, and diluted in absolute methanol. Haemolymph pigment samples were transferred into 1.5 mL cuvettes and the absorbance spectra were acquired using a Shimadzu UV-1800 UV/Visible Scanning Spectrophotometer. Spectral data was analysed using Shimadzu UVProbe and R Software.

### Imaging of adult phenotypes

Adults were frozen for one day at − 20 °C. Butterfly wings were removed using a pair of fine scissors and imaged under a Leica DMS1000 microscope. For imaging of adult eyes, adults were frozen for 40 min at − 20 °C in order to minimize the extent of post-mortem compound eye color changes prior to imaging.

### RT-PCR

Pupal wing tissue was dissected from wild-type individuals 15% PD (24 h) and stored in Invitrogen™ RNAlater™ Stabilization Solution. Total RNA was extracted and purified using the Qiagen RNeasy Plus Micro Kit (Cat No. 74034). Complementary DNA was synthesised using Invitrogen™ SuperScript™ II Reverse Transcriptase (Cat No. 18064014) and diluted to 50 ng/μL. PCR was conducted on the cDNA using primers specific to the genes of interest and reference gene *ef1α* (Table S4) using 2× PCRBIO Taq Mix Red (Cat. no.: PB10.11-20) for 32 cycles. The PCR products were run on an 1% agarose gel and imaged under a gel documentation system (Azure 200 Gel Imaging System: AZI200).

### Scanning electron microscopy (SEM)

A fine metal needle was used to pick scales from adult WT wings. The scales were then mounted on a carbon tape fixed to an SEM stub, platinum coated using JEOL JFC-1600 Auto Fine Coater and imaged under a JEOL JSM-6701F Field-Emission SEM.

## Supplementary Information

Supplementary Information.

## Data Availability

The datasets analysed in the current study are available in the NCBI repository. All the sequences used along with their gene IDs and direct link are mentioned in the supplementary file.
