# Author Correction: *Vermilion* and *cinnabar* are involved in ommochrome pigment biosynthesis in eyes but not wings of *Bicyclus anynana* butterflies

**DOI:** 10.1038/s41598-023-37368-7

**Published:** 2023-06-22

**Authors:** Shaun Hong Chuen How, Tirtha Das Banerjee, Antόnia Monteiro

**Affiliations:** Department of Biological Sciences, National University of Singapore, Singapore, 117557 Singapore

Correction to: *Scientific Reports* 10.1038/s41598-023-36491-9, published online 09 June 2023

The original version of this Article contained an error in the name of author Shaun Hong Chuen How, which was incorrectly given as How Hong Chuen Shaun.

The original Article and accompanying Supplementary Information file have been corrected.

