# Supplementary material for: Vermilion and cinnabar are involved in ommochrome pigment biosynthesis in eyes but not wings of Bicyclus anynana butterflies
Source: Sci Rep. 2023 Jun 9;13:9368. doi: 10.1038/s41598-023-36491-9 (PMC10256707; doi:10.1038/s41598-023-36491-9)
Supplement: Supplementary file 1 — Supplementary Information. [file 41598_2023_36491_MOESM1_ESM.docx]

***vermilion* and *cinnabar* are involved in ommochrome pigment biosynthesis in eyes but not wings of *Bicyclus anynana* butterflies**

**Shaun Hong Chuen How^1,#^, Tirtha Das Banerjee^1,#,*^, and Antόnia Monteiro^1,*^**

**Affiliation**

1. Department of Biological Sciences, National University of Singapore, Singapore, 117557.

# These authors contributed equally

* Authors for correspondence: [tirtha_banerjee@u.nus.edu](mailto:tirtha_banerjee@u.nus.edu); [antonia.monteiro@nus.edu.sg](mailto:antonia.monteiro@nus.edu.sg)

**
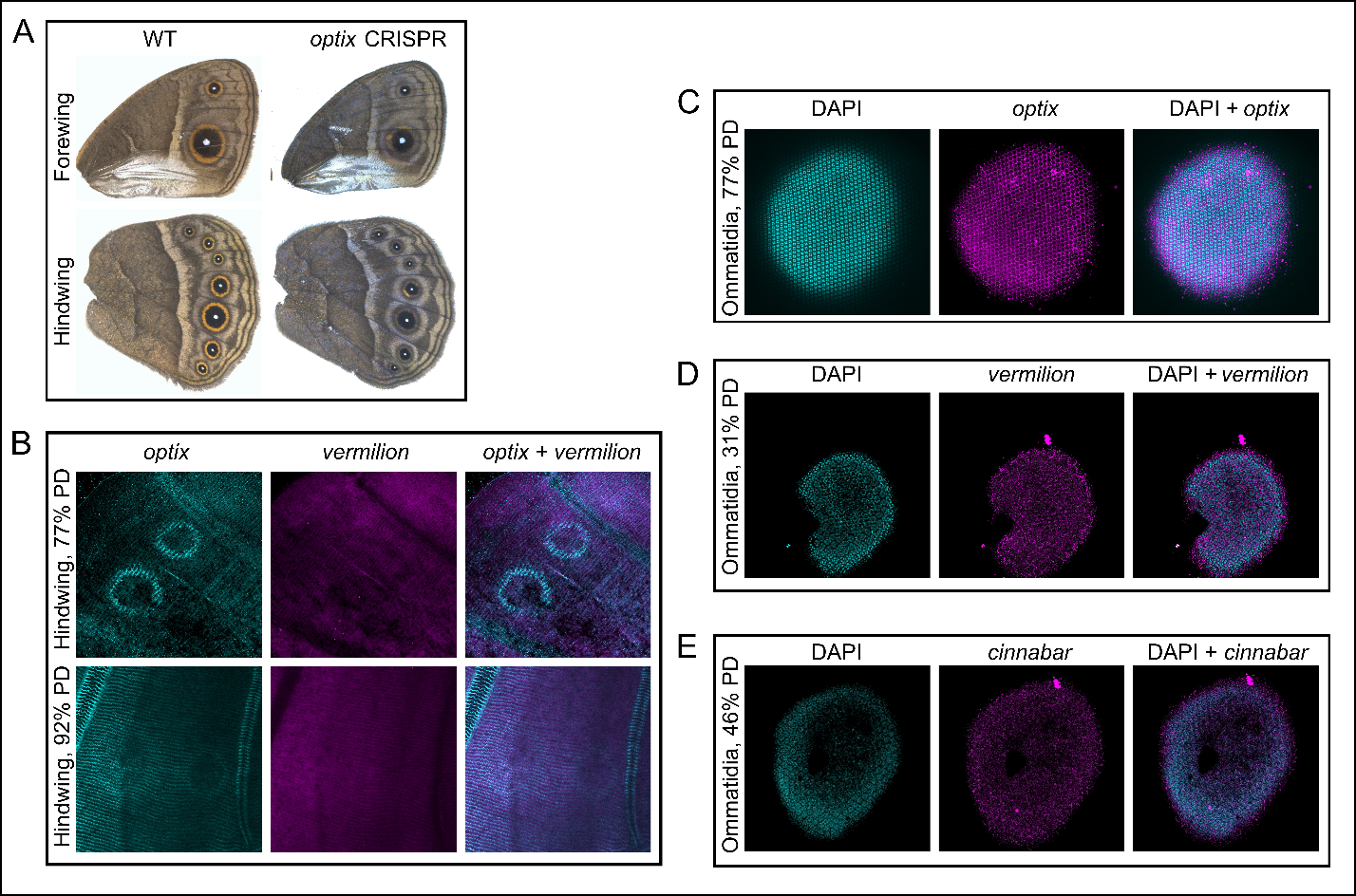
Figure S1. Function of *optix* in adult wings of *Bicyclus anynana* and expression of *optix* and *vermilion* in pupal wings at 77% and 92% PD (120 hrs and 144 hrs), and in ommatidia at 31% and 77% PD (48 hrs and 120 hrs).** (A) WT adult wing and *optix* CRISPR showing defects in pigmentation in the wings. (B) Co-expression of *optix* and *vermilion* in pupal wings at 77 and 92% PD (120 and 144 hrs) using HCR. *optix* is expressed in the orange ring cells of the eyespot while no specific domain of expression for *vermilion* is observed. (C) Expression of *optix* along with DAPI in ommatidia at 77% PD (120 hrs). (D) Expression of *vermilion* and DAPI in ommatidia at 31% PD (48 hrs). (E) Expression of *cinnabar* and DAPI in ommatidia at 46% PD (72 hrs).


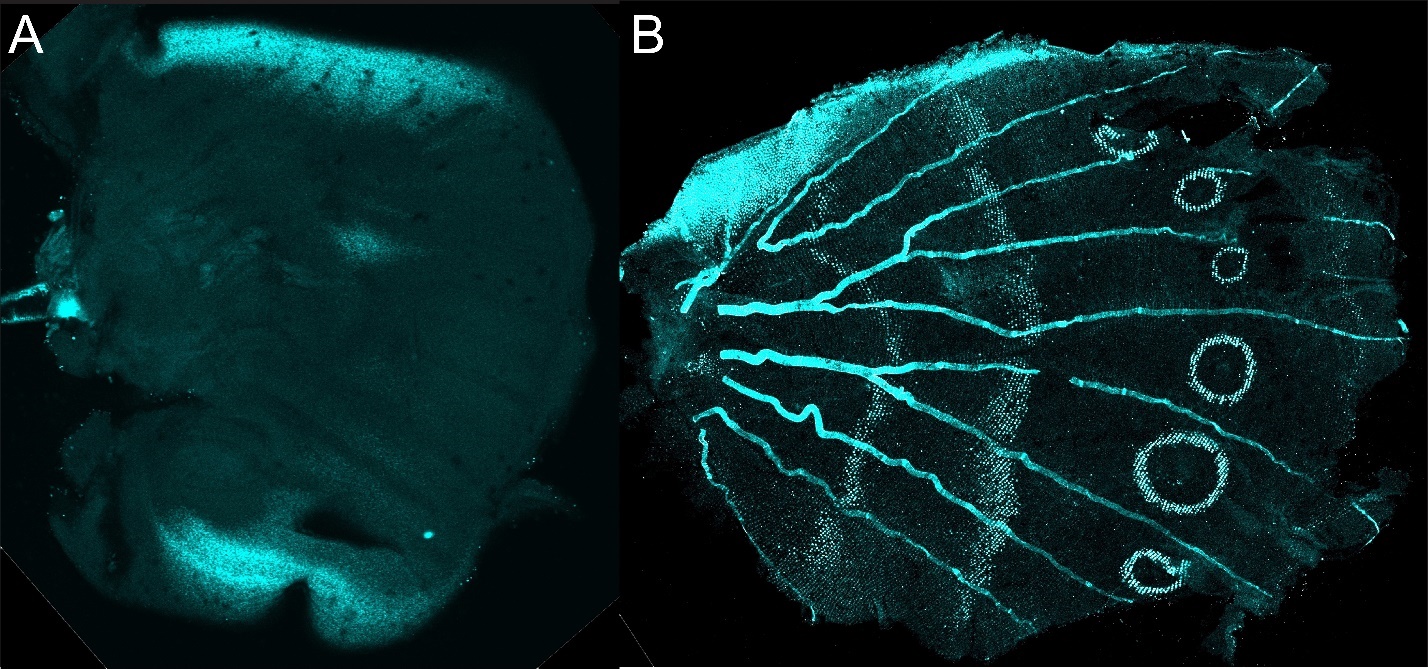


**Figure S2. Expression of *optix* in the mid larval and 31% PD (48 hrs) pupal hindwing of *B. anynana*. (A)** During larval wing development *optix* is expressed strongly in the upper anterior and the lower posterior compartment. (**B**) During the pupal stage, *optix* shows expression in the anterior silver scale region, along the edges of the central symmetry system, the media I and media II bands, in the eyespot orange ring, and along the externa I.

**
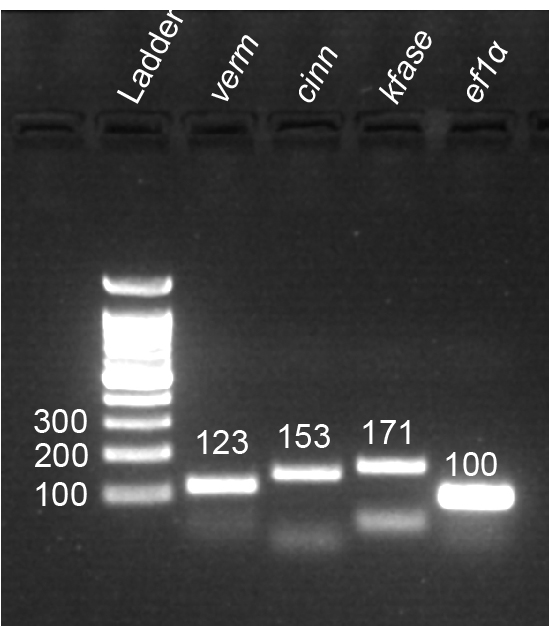
**

**Figure S3. cDNA based verification of *vermilion (verm)*, *cinnabar (cinn), kfase*, and reference gene *ef1α* in the 24 hrs pupal wings of *B. anynana*.** Amplicons of *verm* (123 bps), *cinn* (153 bps), *kfase* (171 bps), and *ef1α* (100 bps). The result shows that the genes are expressed at lower levels in the pupal wings relative to the reference.


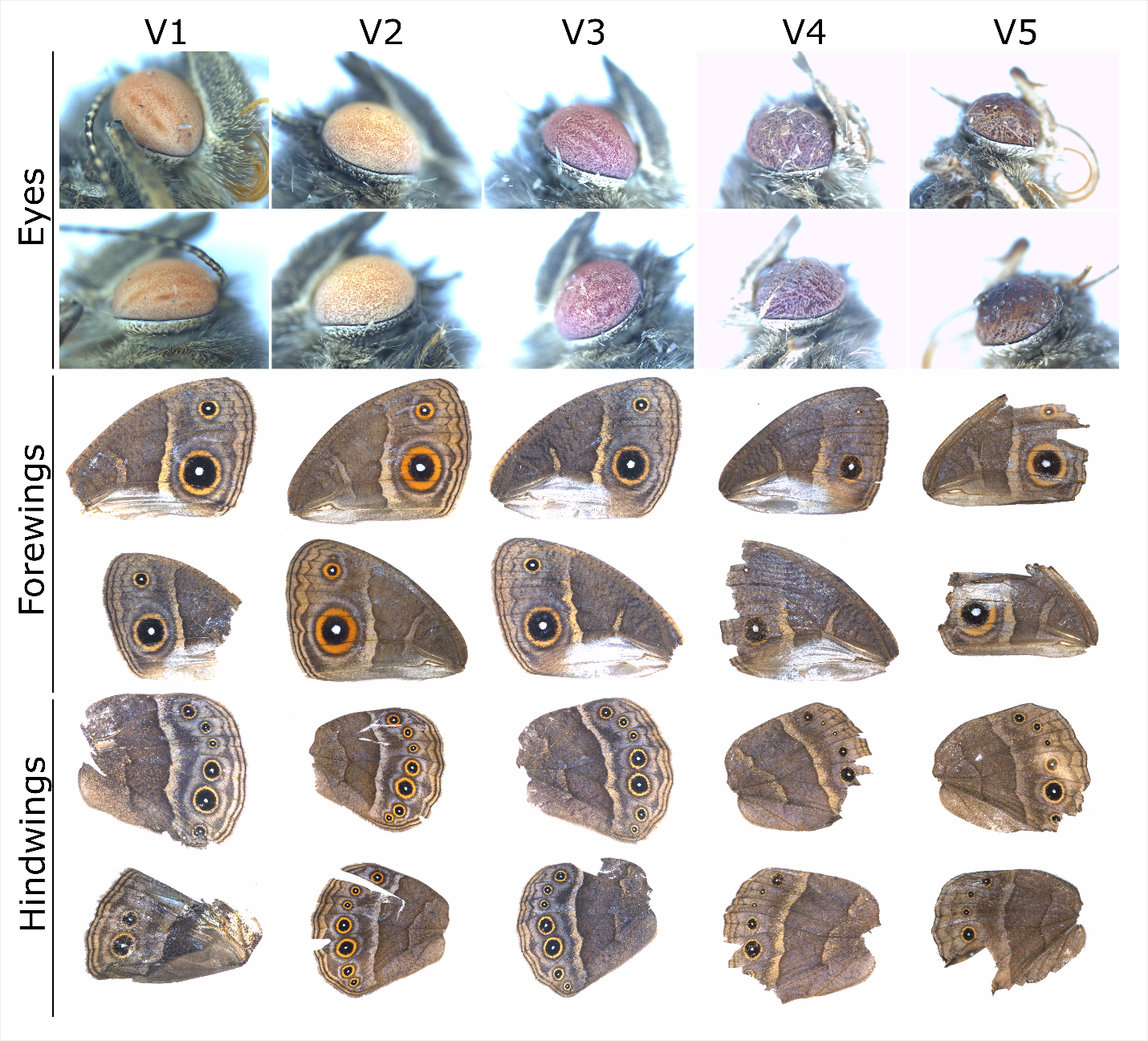


**Figure S4. Eye and wing phenotype of *vermilion* CRISPR mutants.** *vermilion* CRISPR results in altered eye pigmentation in mosaic clones. No wing phenotype was observed.


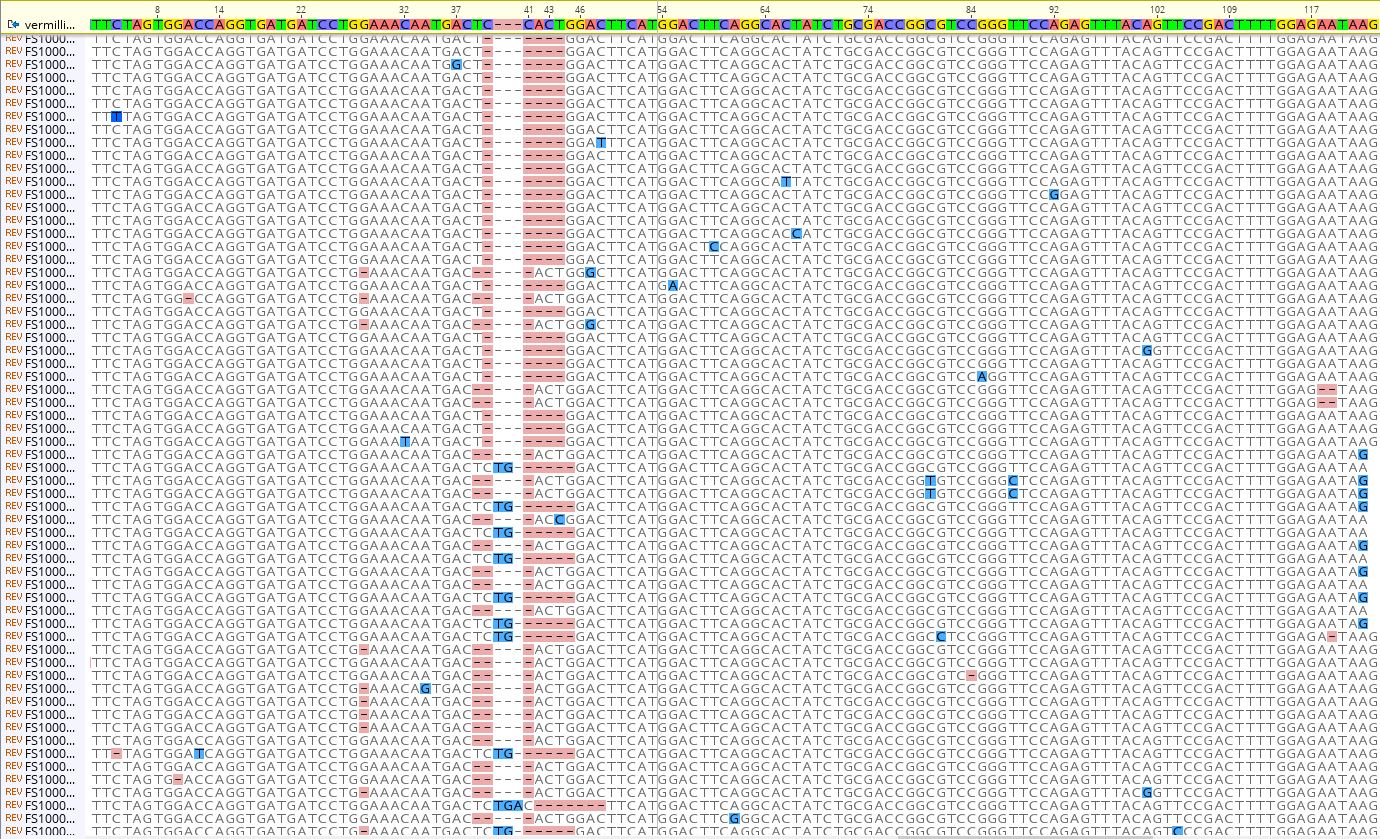


**Figure S5. Indels from *vermilion* eye CRISPR (V1) tissue showing deletions at the target site.**


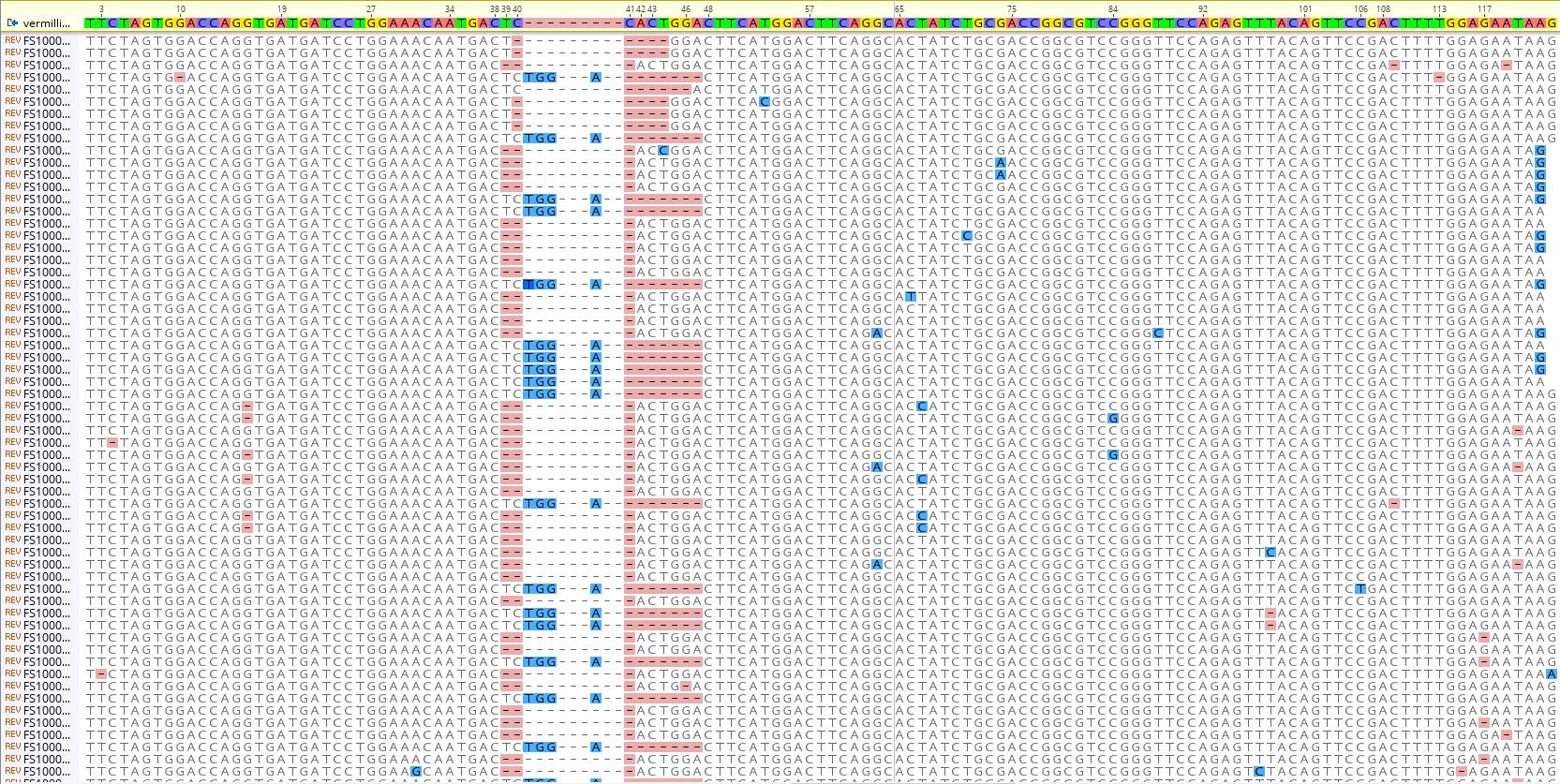
 **Figure S6. Indels from *vermilion* eye CRISPR (V2) tissue showing deletions at the target site.**


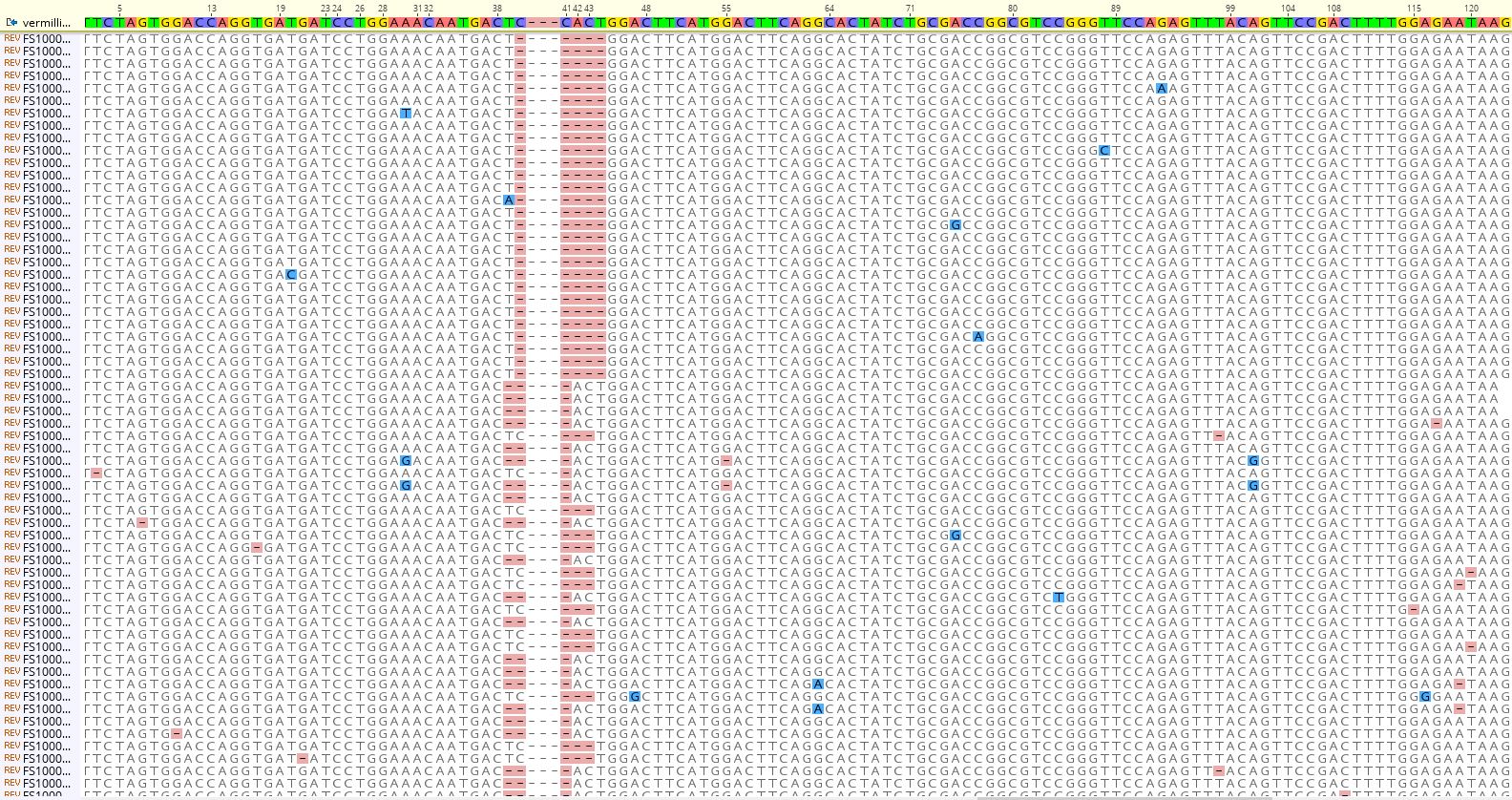


**Figure S7. Indels from *vermilion* eye CRISPR (V3) tissue showing deletions at the target site.**


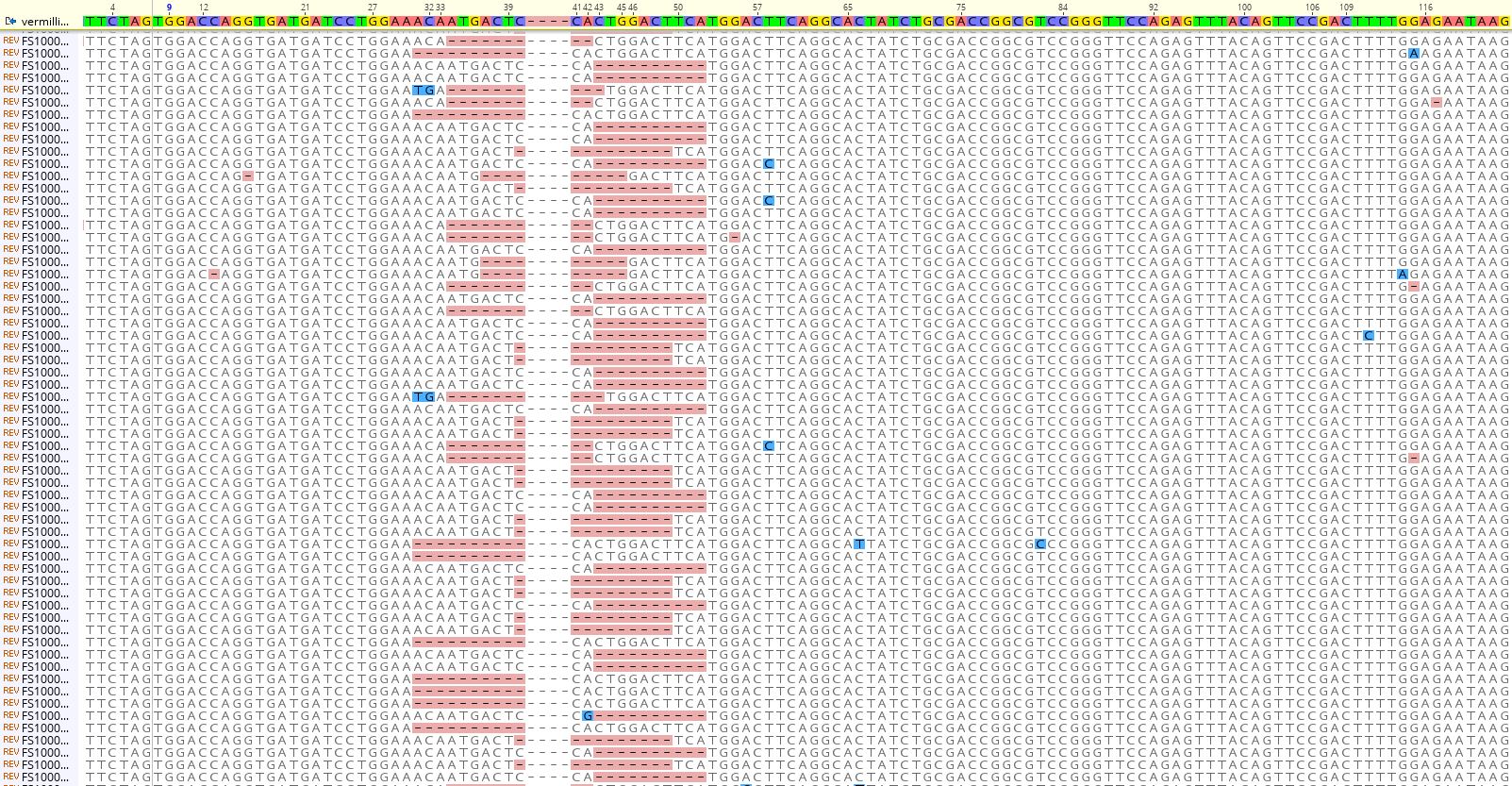


**Figure S8. Indels from *vermilion* eye CRISPR (V4) tissue showing deletions at the target site.**


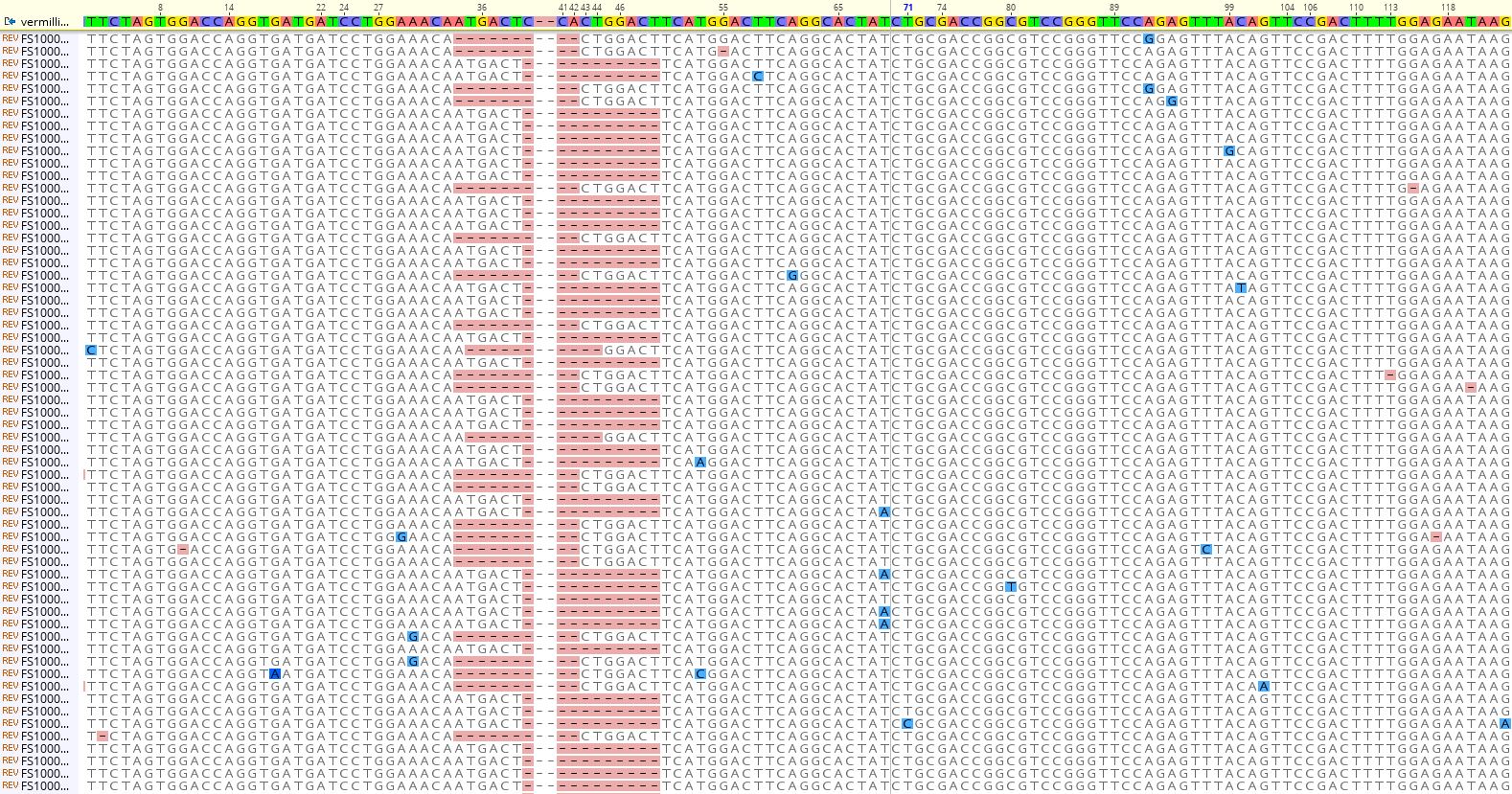


**Figure S9. Indels from *vermilion* eye CRISPR (V5) tissue showing deletions at the target site.**


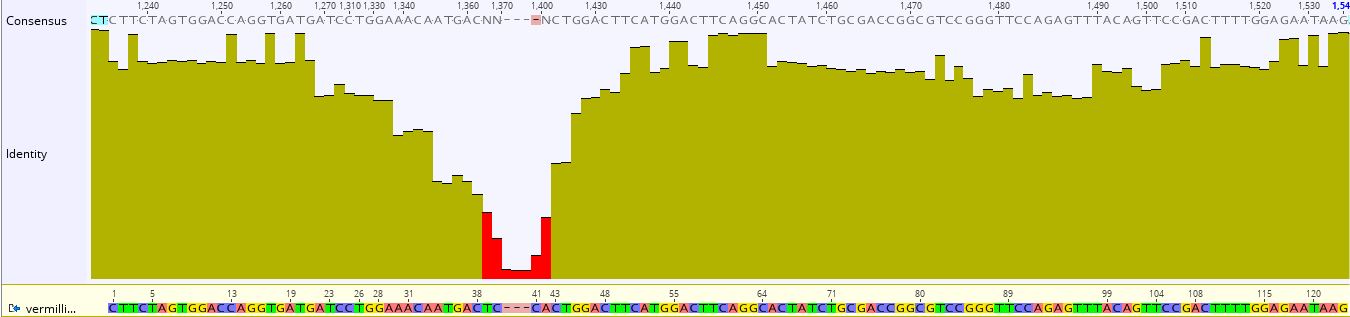

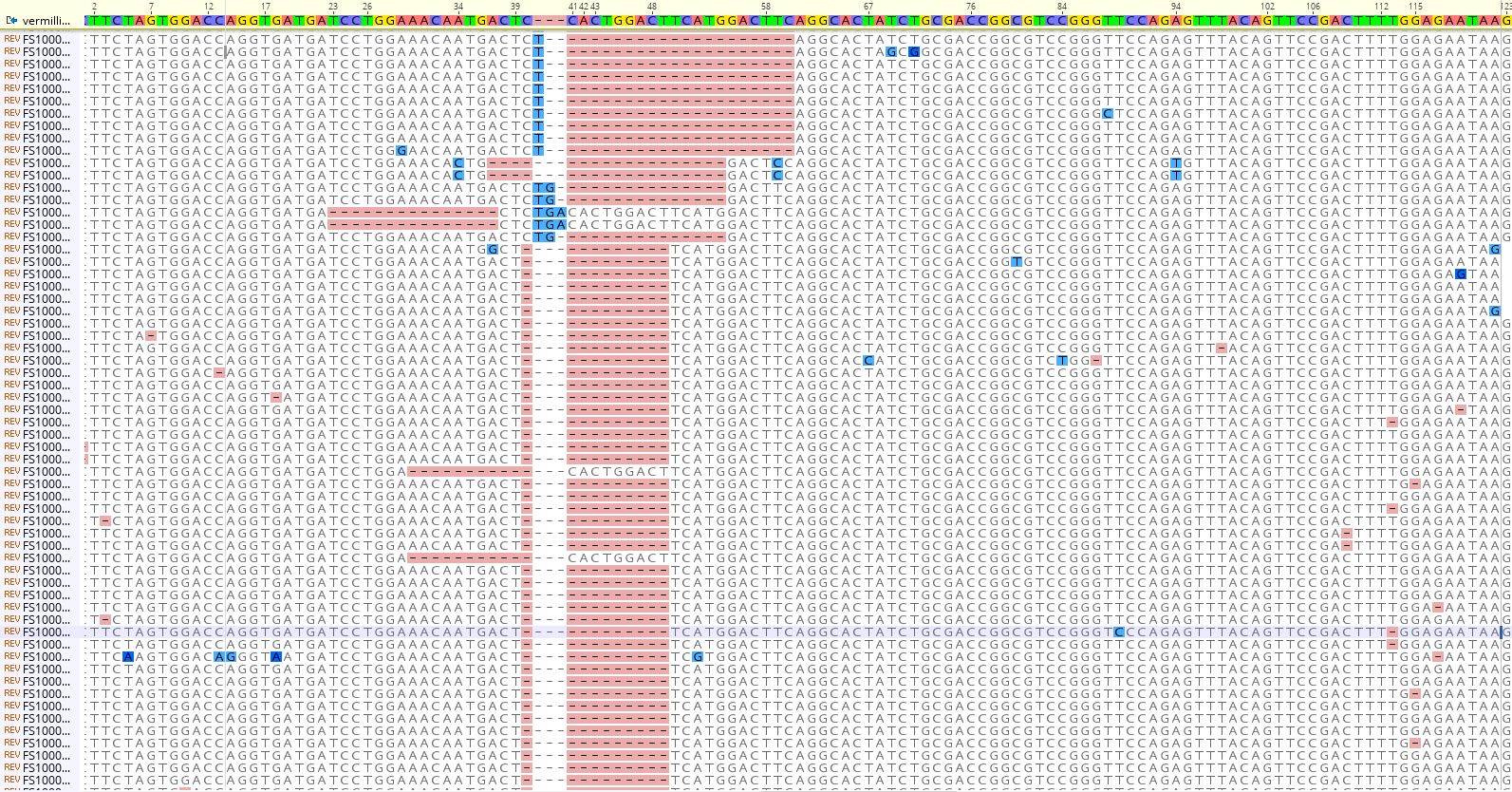


**Figure S10. Mean pairwise identity of all *vermilion* wing CRISPR (V1) tissue reads against reference (greenish-brown denotes 30-99% identity, red denotes <30% identity). Indels showing deletions at the target site.**


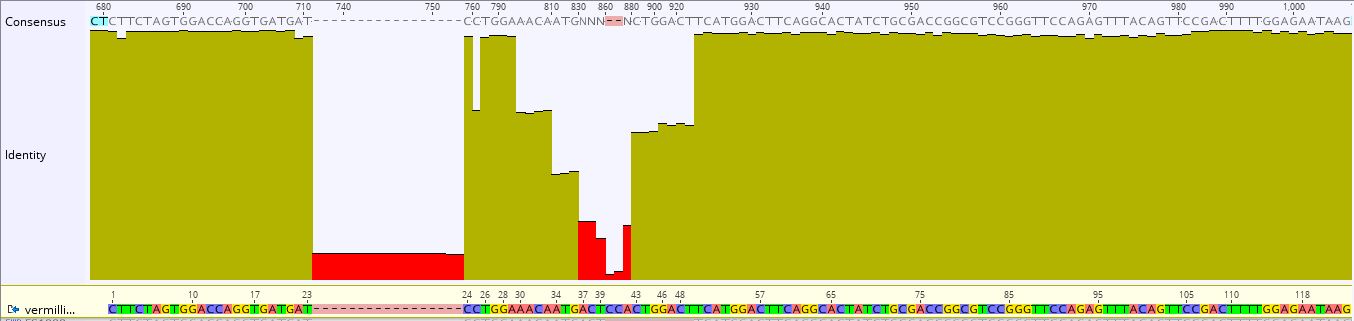

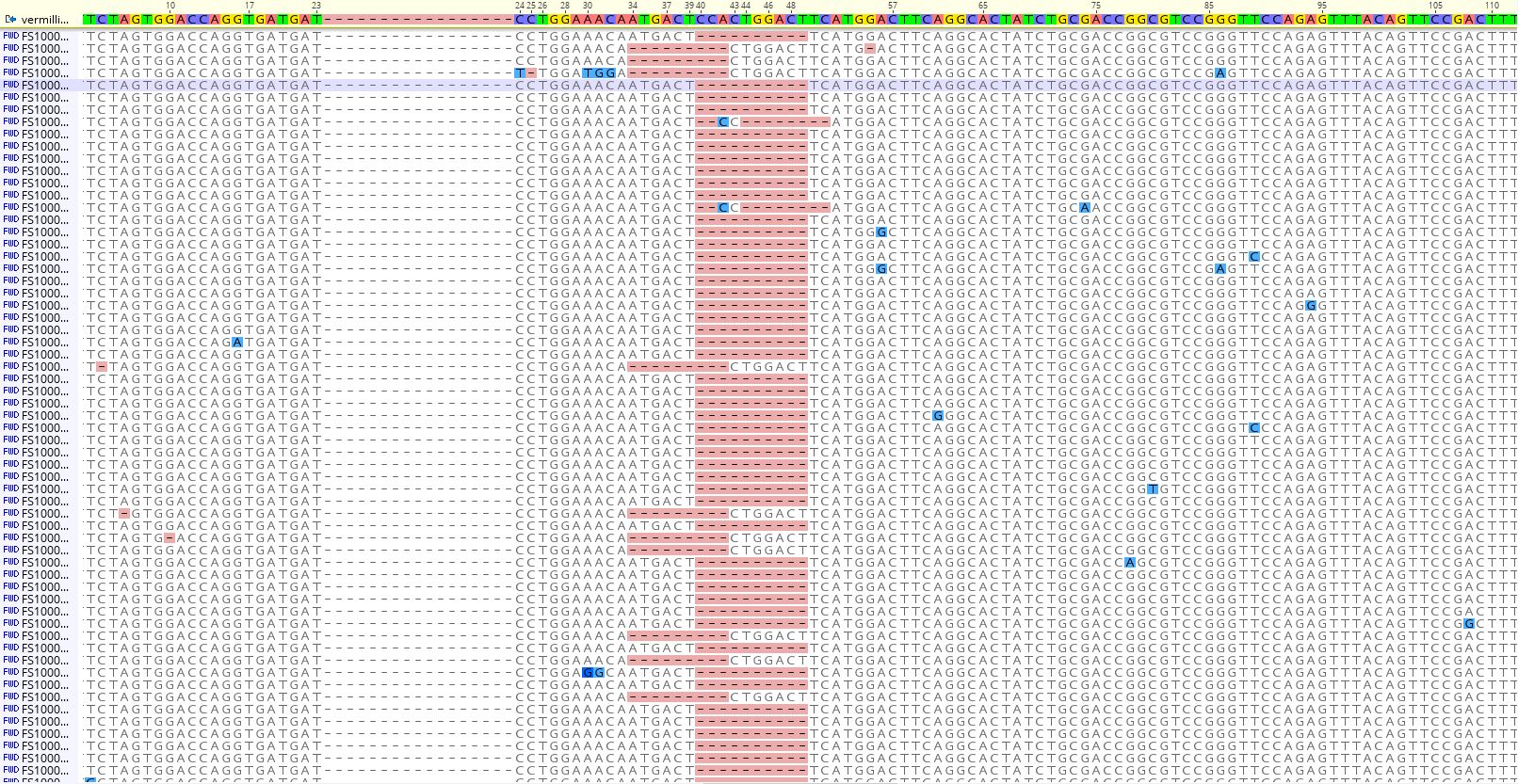


**Figure S11. Mean pairwise identity of all *vermilion* wing CRISPR (V2) tissue reads against reference (greenish-brown denotes 30-99% identity, red denotes <30% identity). Indels showing deletions at the target site.**


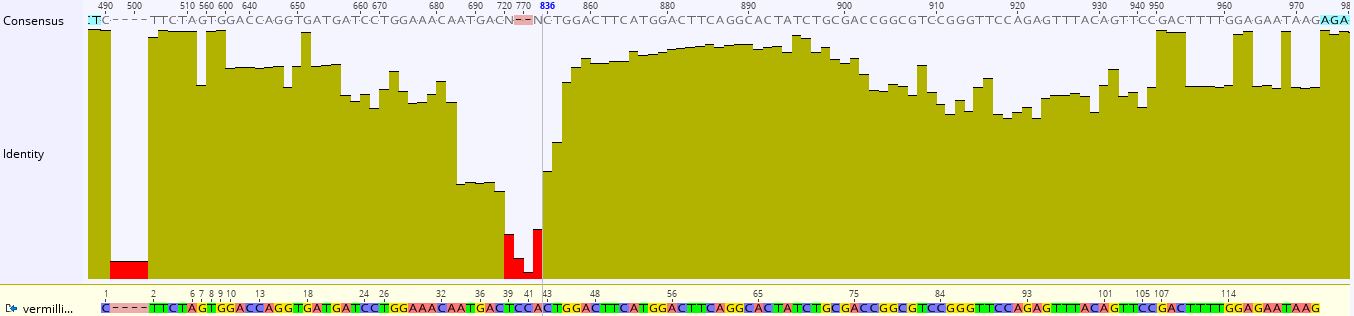

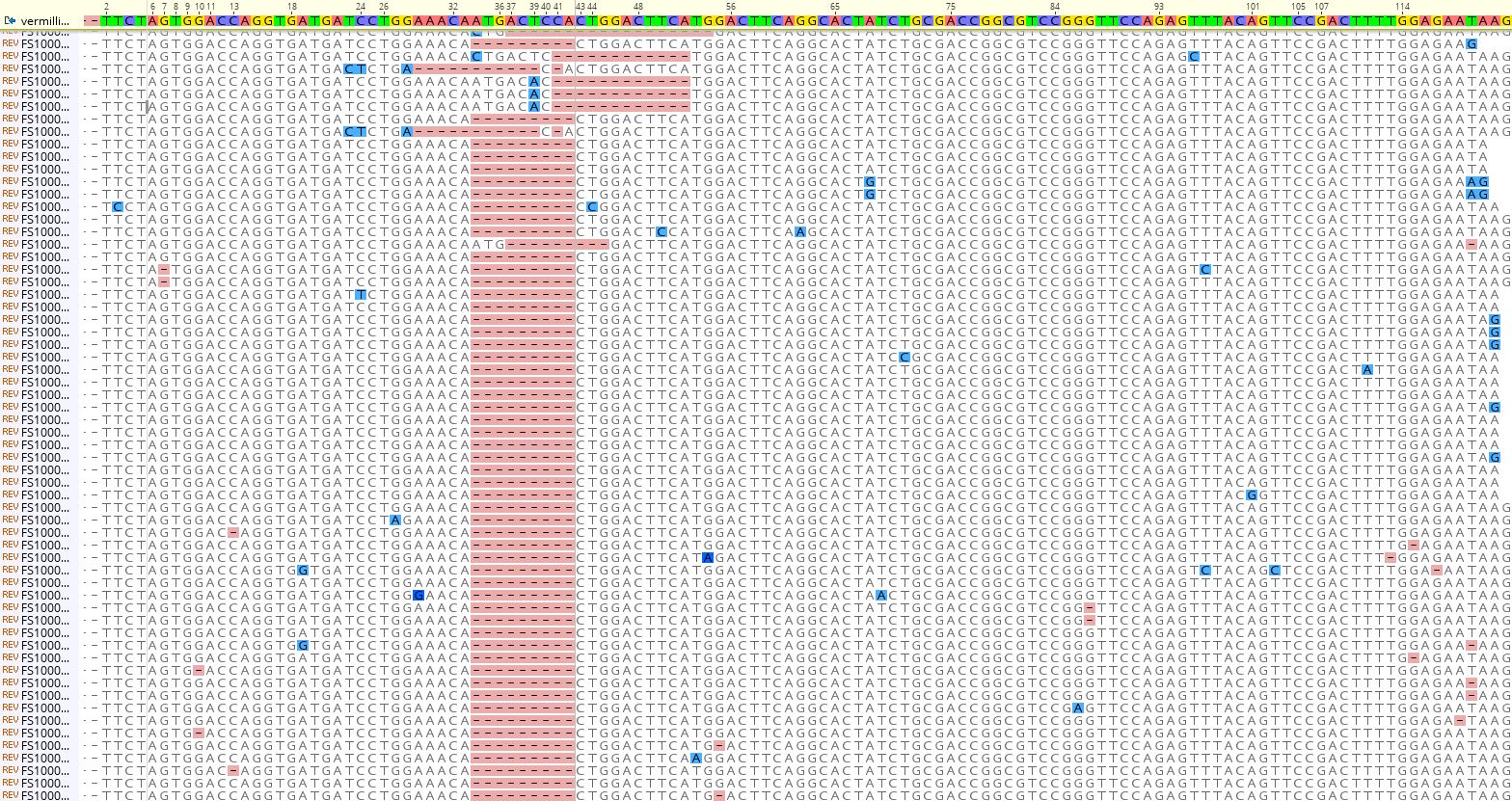


**Figure S12. Mean pairwise identity of all *vermilion* wing CRISPR (V3) tissue reads against reference (greenish-brown denotes 30-99% identity, red denotes <30% identity). Indels showing deletions at the target site.**


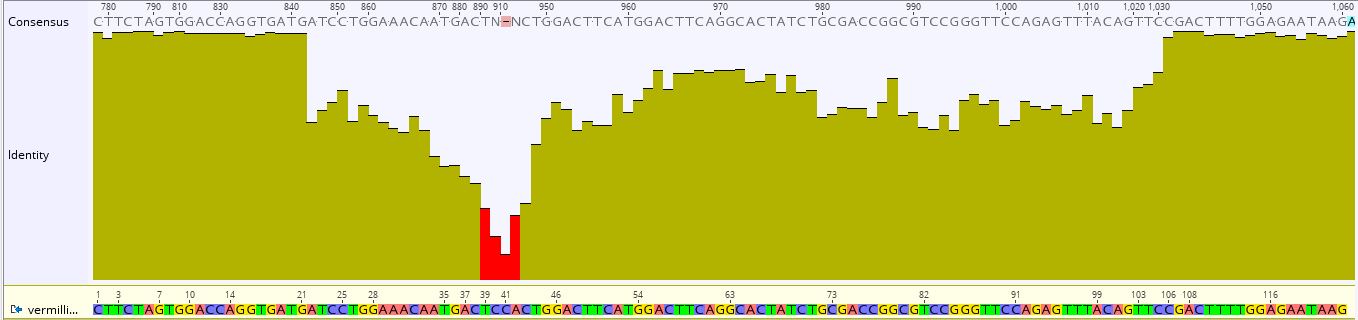

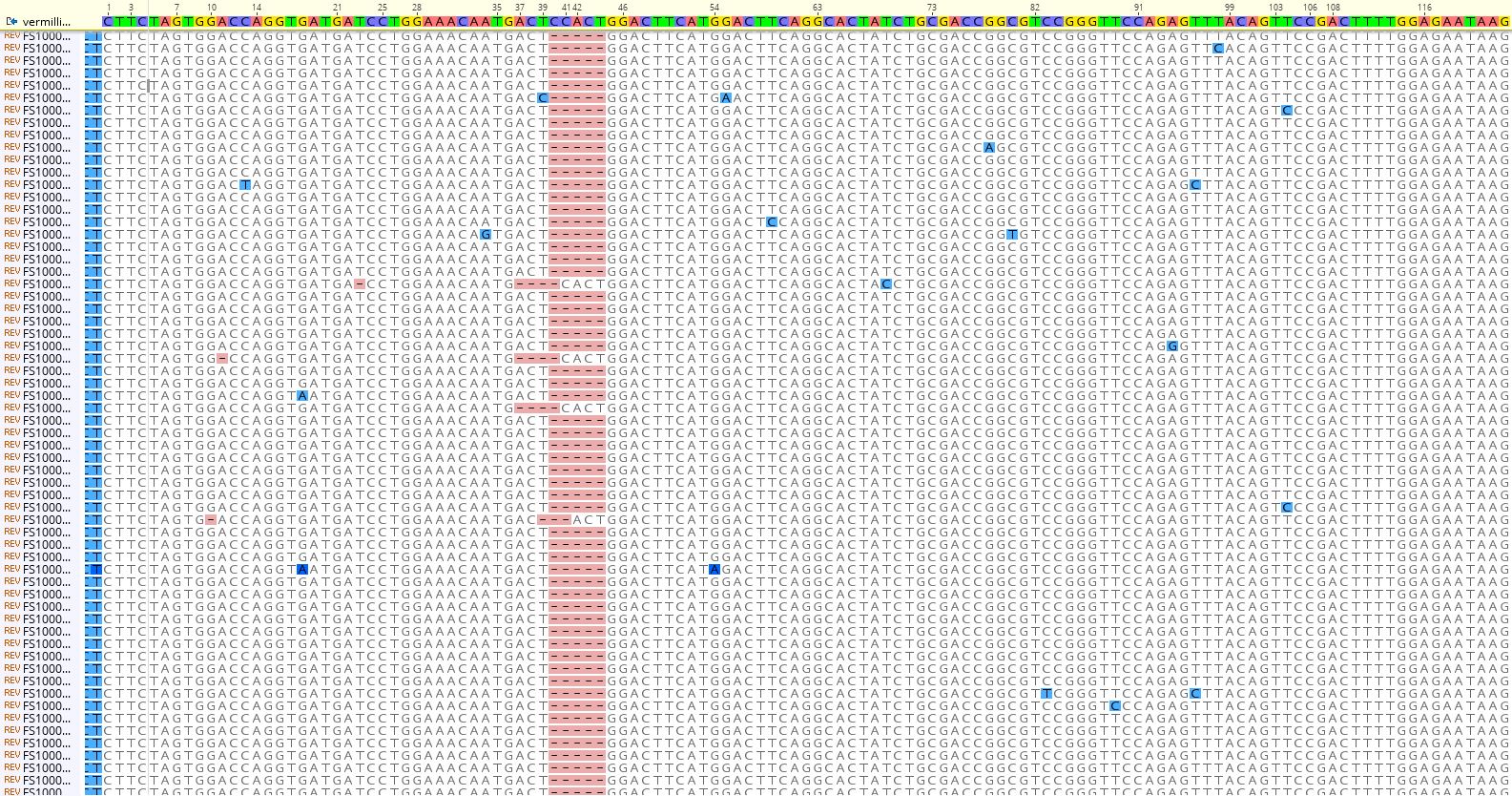


**Figure S13. Mean pairwise identity of all *vermilion* wing CRISPR (V4) tissue reads against reference (greenish-brown denotes 30-99% identity, red denotes <30% identity). Indels showing deletions at the target site.**


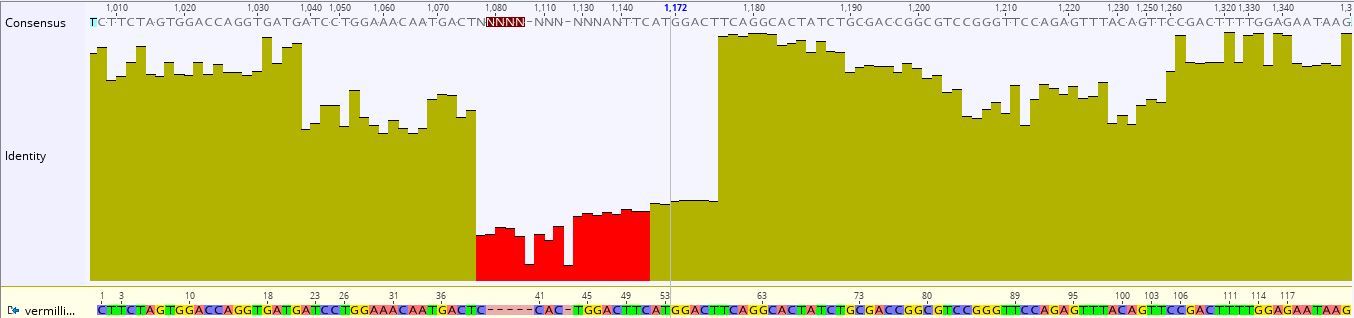

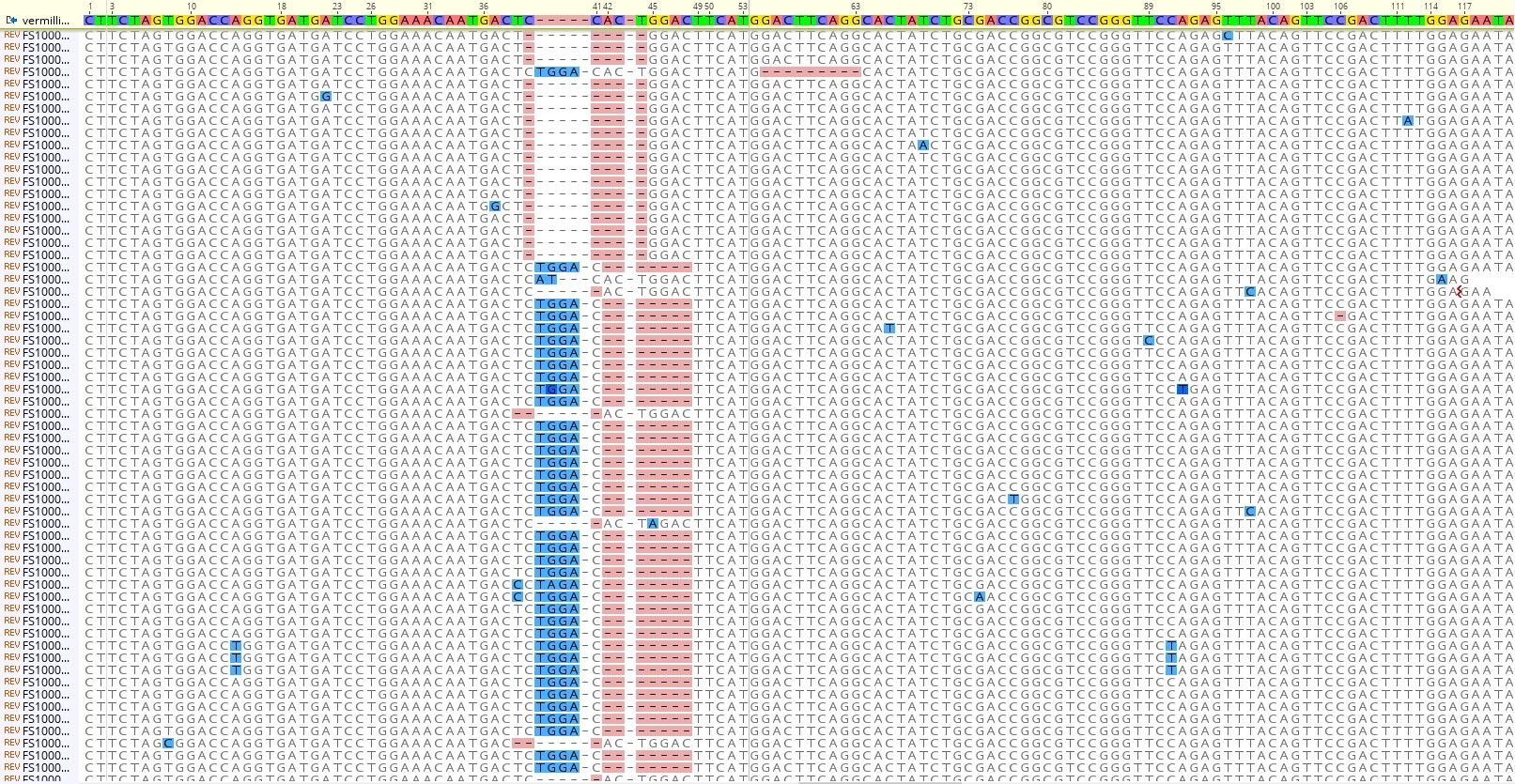


**Figure S14. Mean pairwise identity of all *vermilion* wing CRISPR (V5) tissue reads against reference (greenish-brown denotes 30-99% identity, red denotes <30% identity). Indels showing deletions at the target site.**


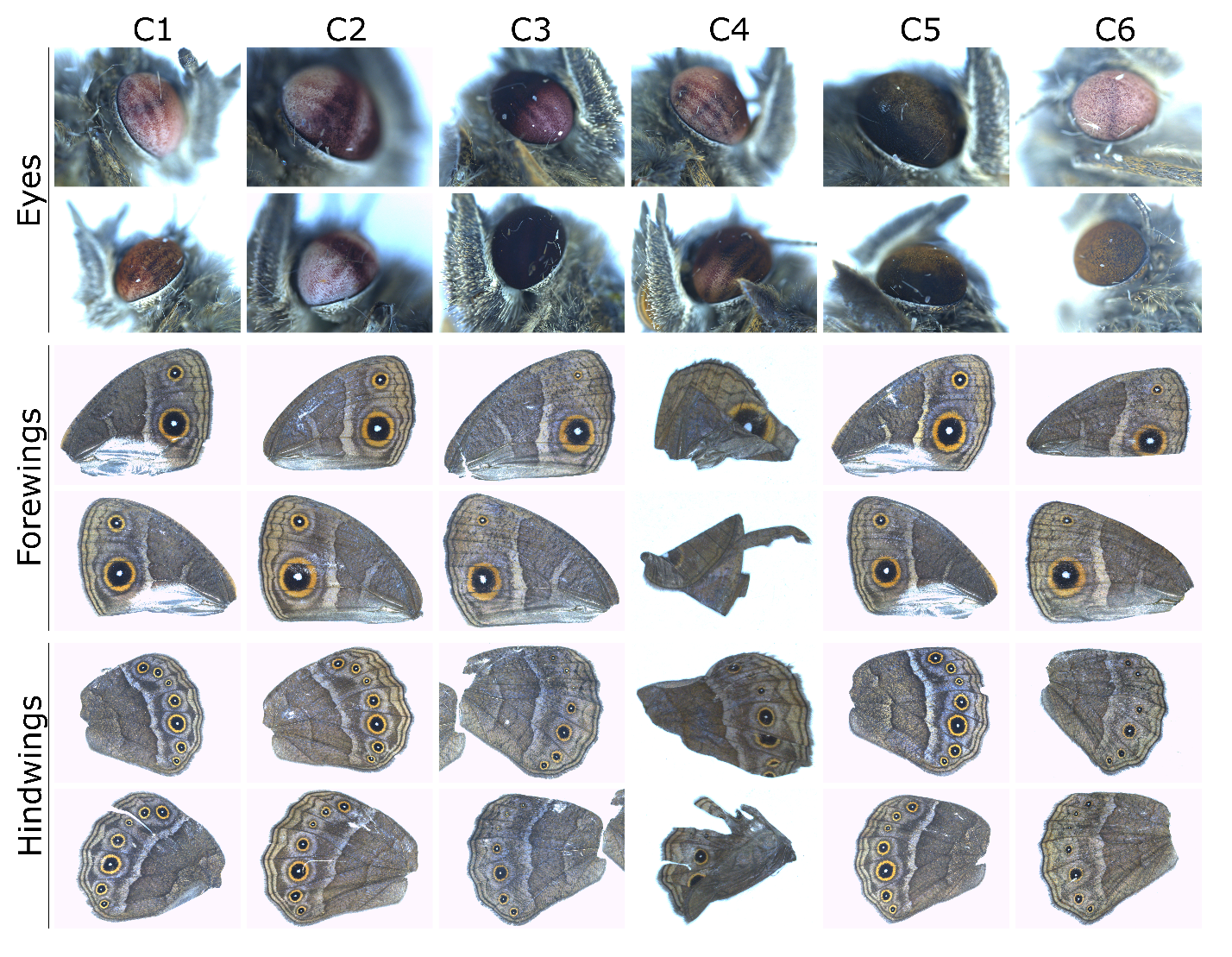


**Figure S15. Eye and wing phenotype of *cinnabar* CRISPR mutants.** *cinnabar* CRISPR results in altered eye pigmentation in mosaic clones. No wing phenotype was observed.


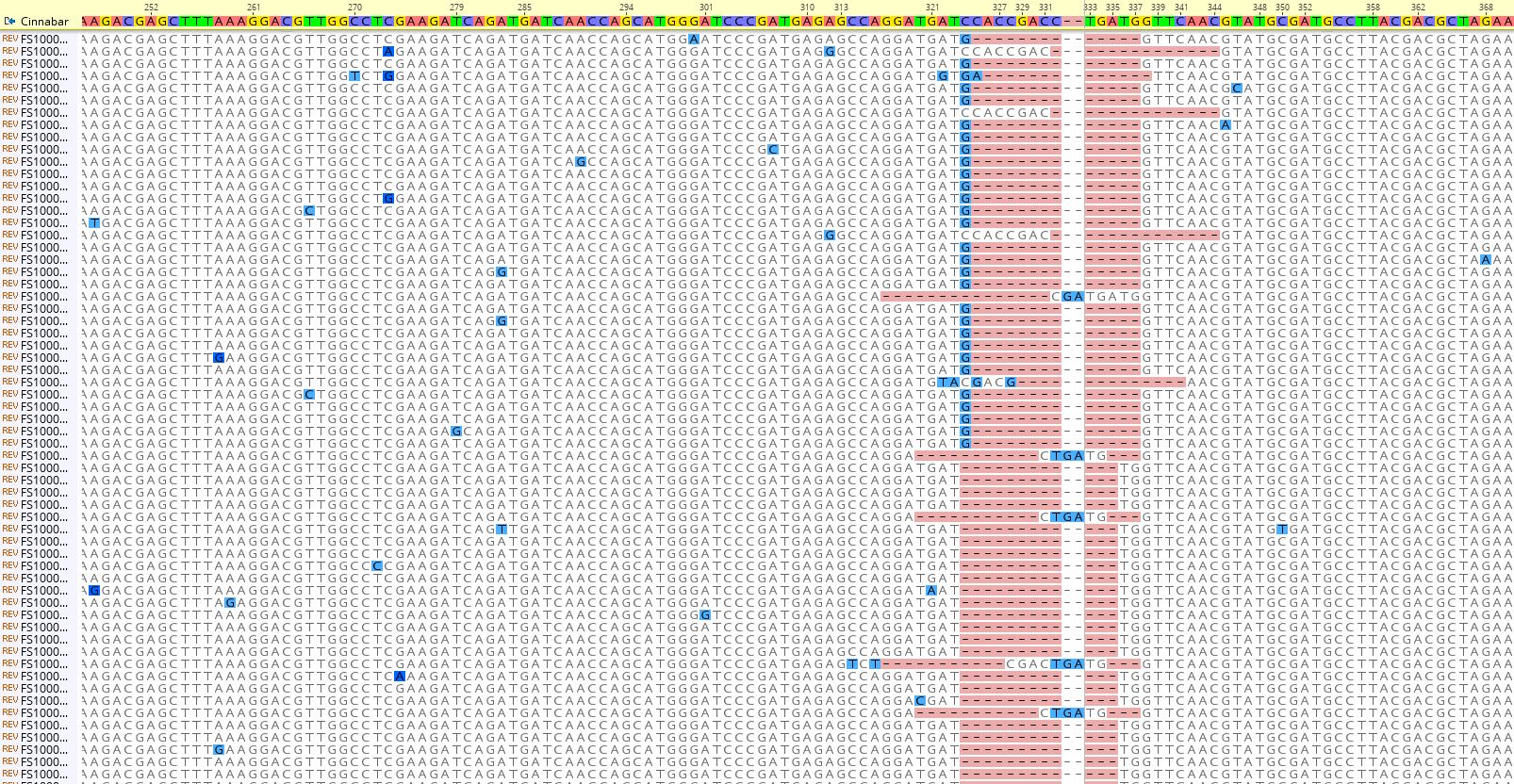


**Figure S16. Indels from *cinnabar* eye CRISPR (C1) tissue showing deletions and insertions at the target site.**


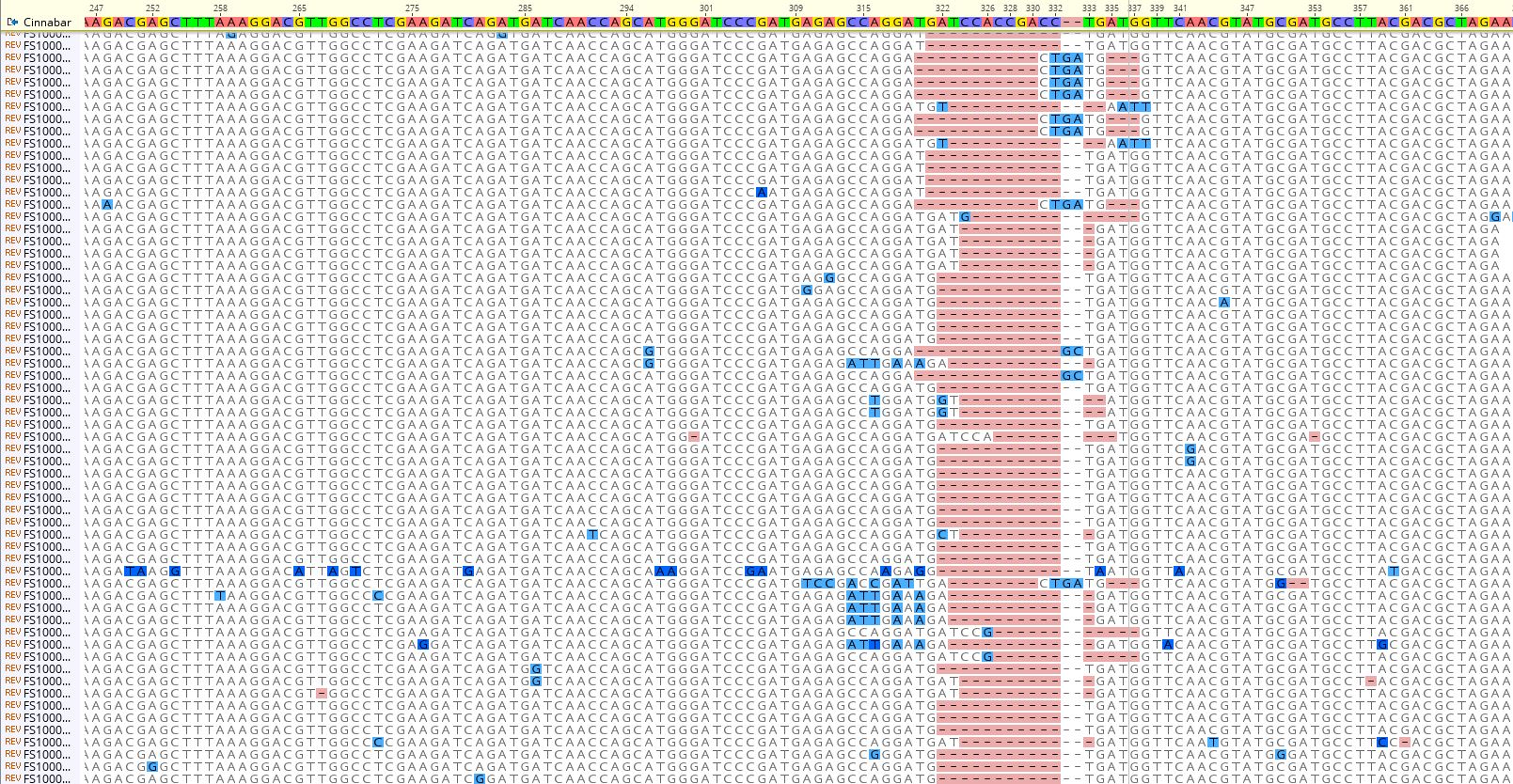


**Figure S17. Indels from *cinnabar* eye CRISPR (C2) tissue showing deletions and insertions at the target site.**


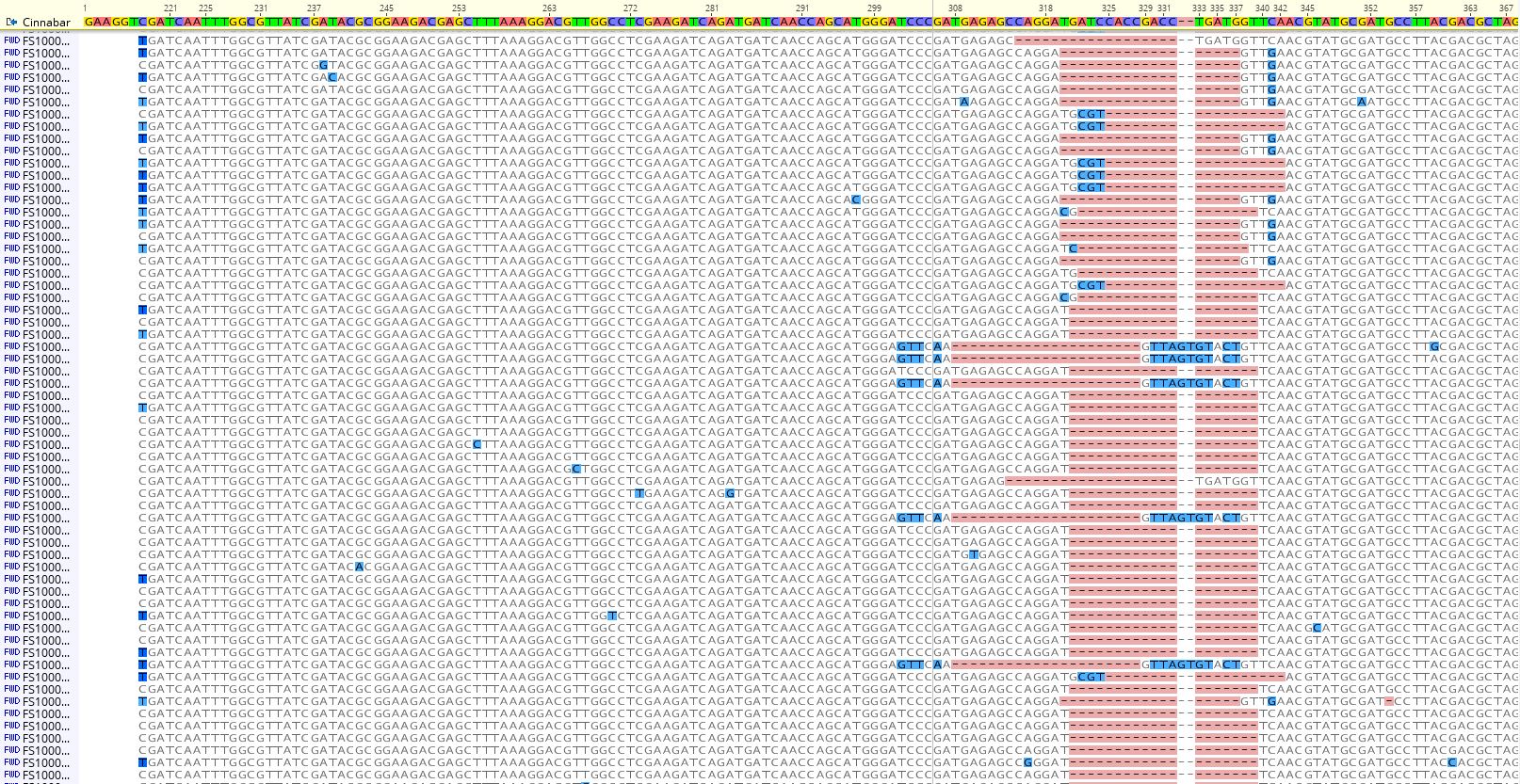


**Figure S18. Indels from *cinnabar* eye CRISPR (C5) tissue showing deletions and insertions at the target site.**


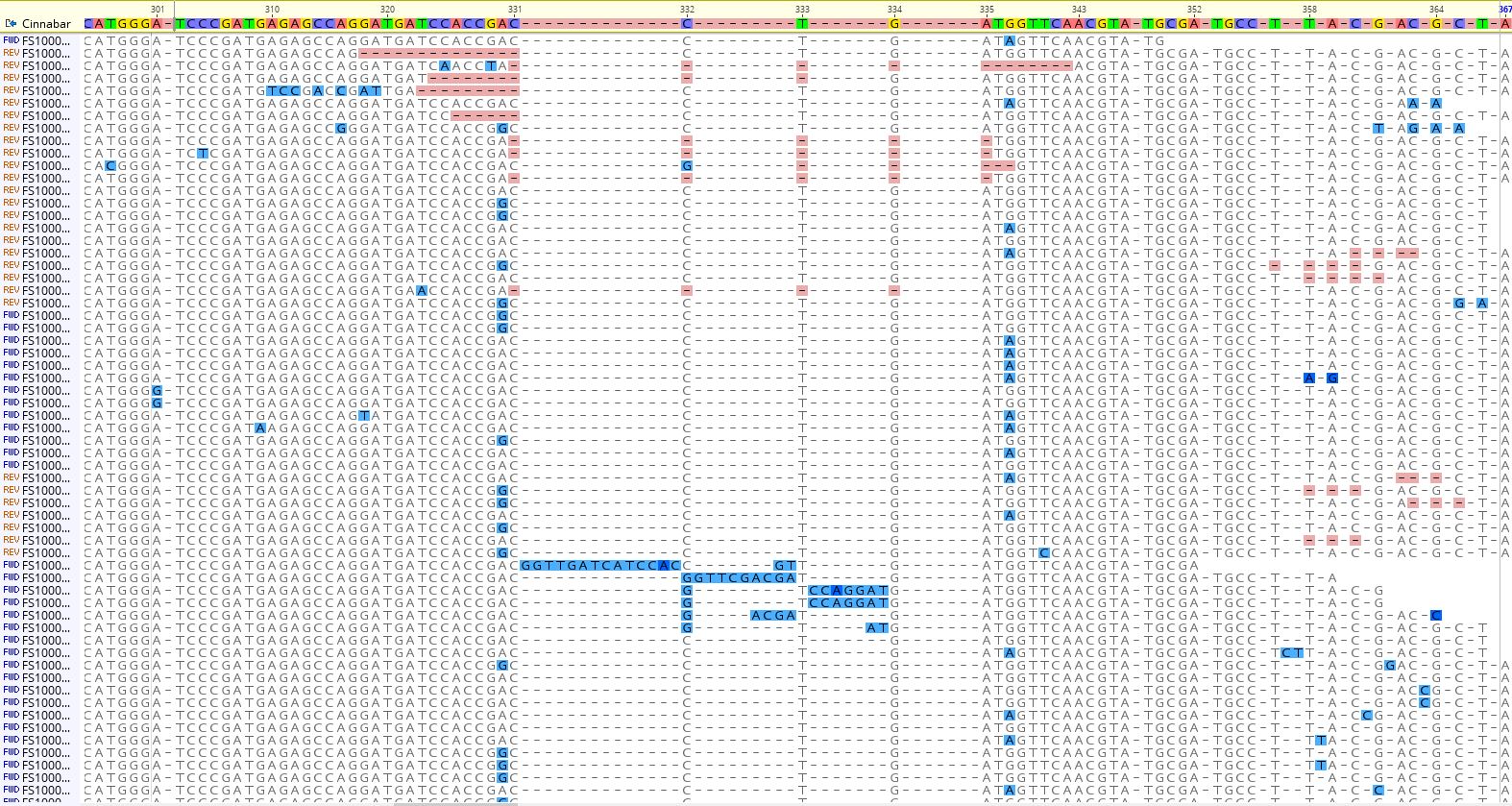


**Figure S19. Indels from *cinnabar* eye CRISPR (C6) tissue showing deletions and insertions at the target site.**


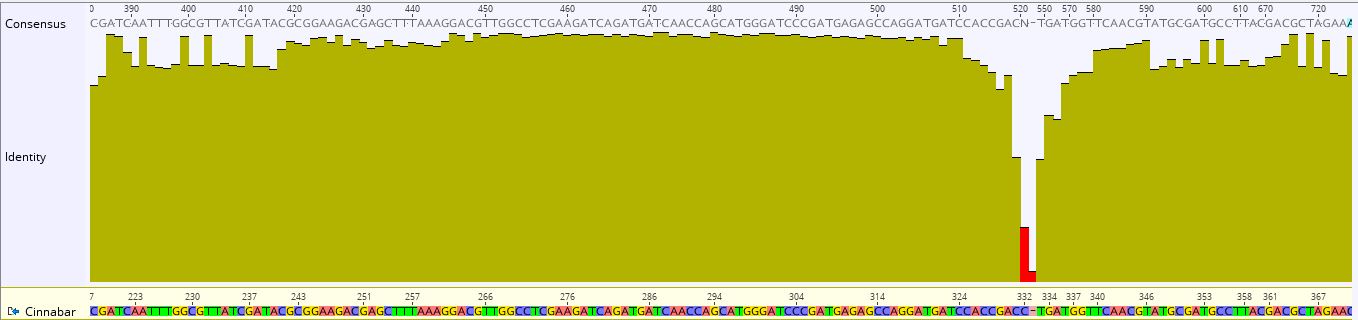

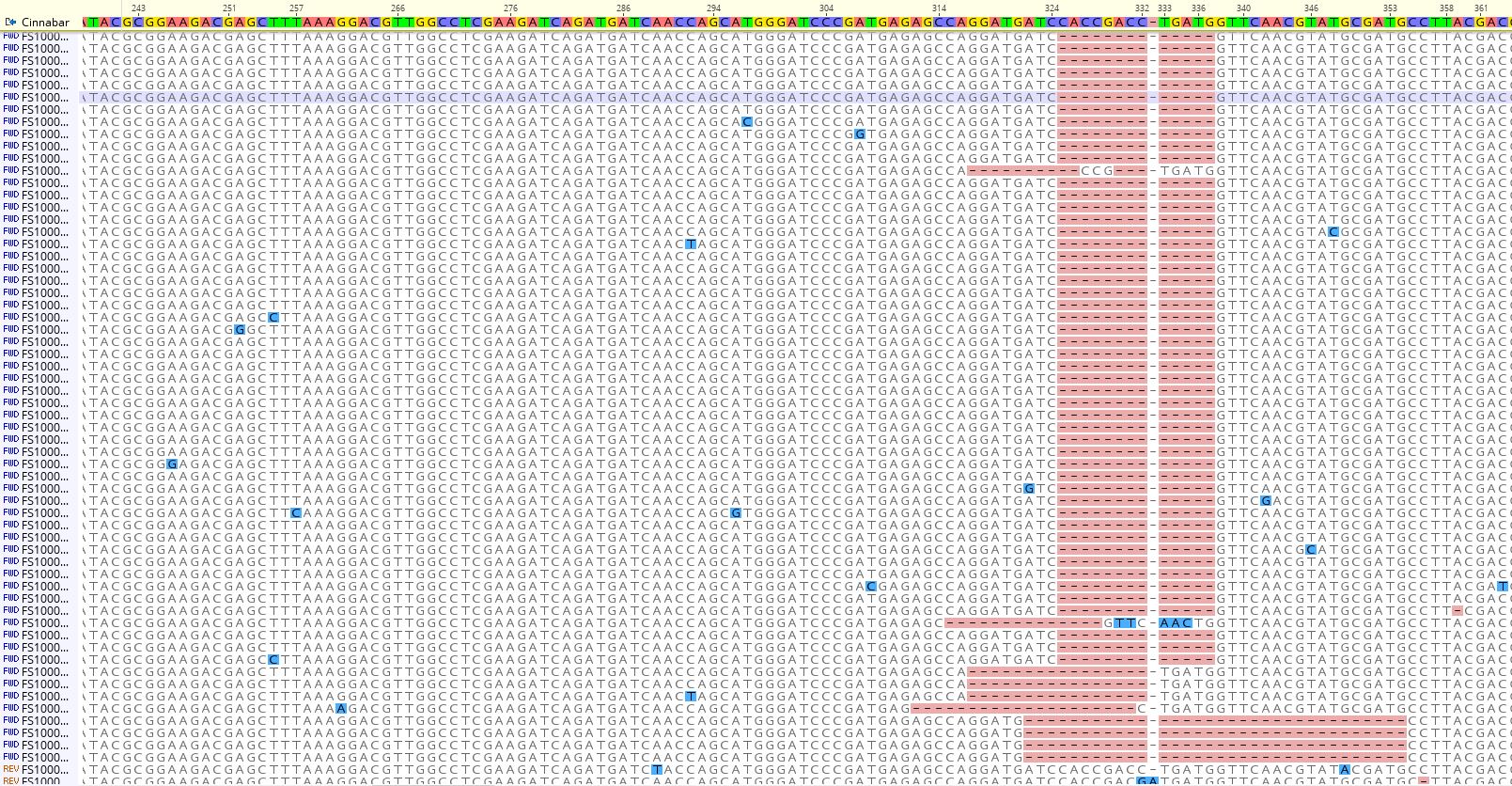


**Figure S20. Mean pairwise identity of all *cinnabar* wing CRISPR (C1) tissue reads against reference (greenish-brown denotes 30-99% identity, red denotes <30% identity). Indels showing deletions at the target site.**


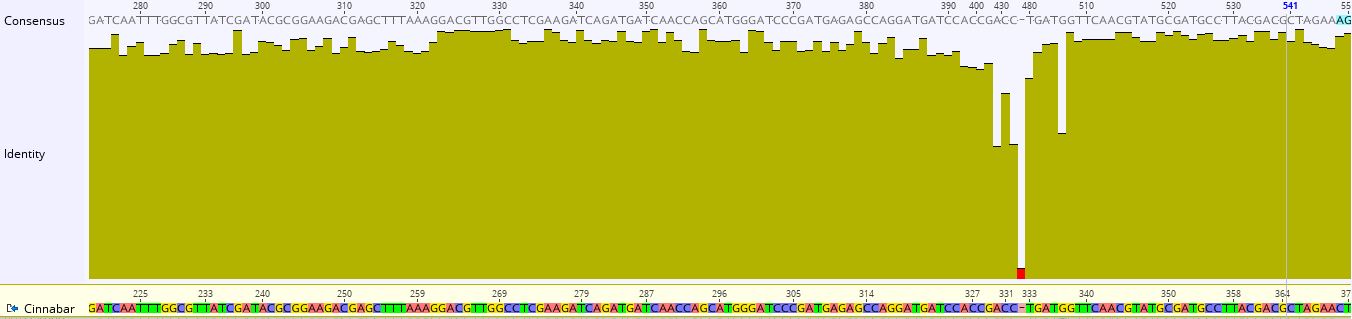

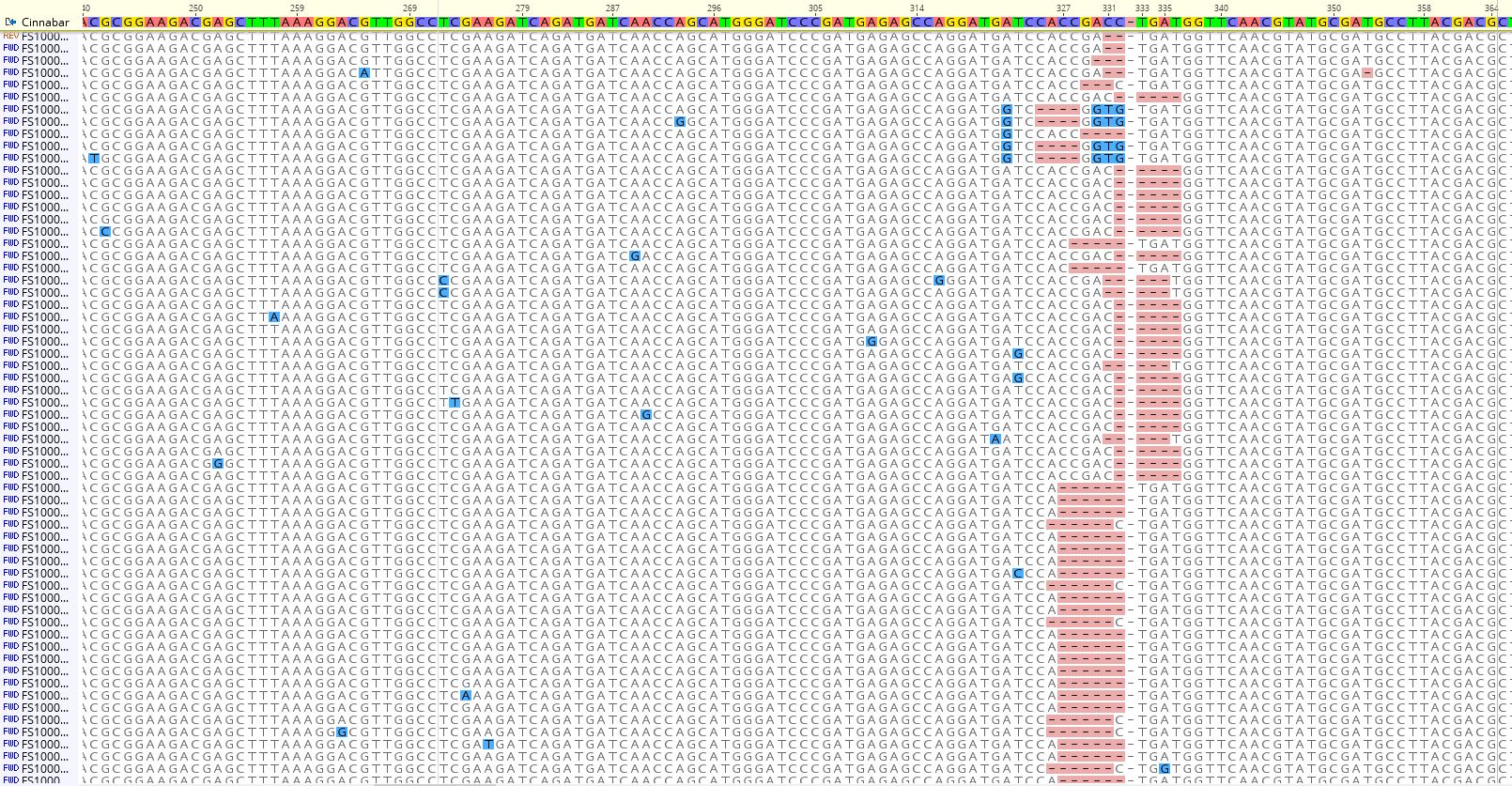


**Figure S21. Mean pairwise identity of all *cinnabar* wing CRISPR (C2) tissue reads against reference (greenish-brown denotes 30-99% identity, red denotes <30% identity). Indels showing deletions at the target site.**


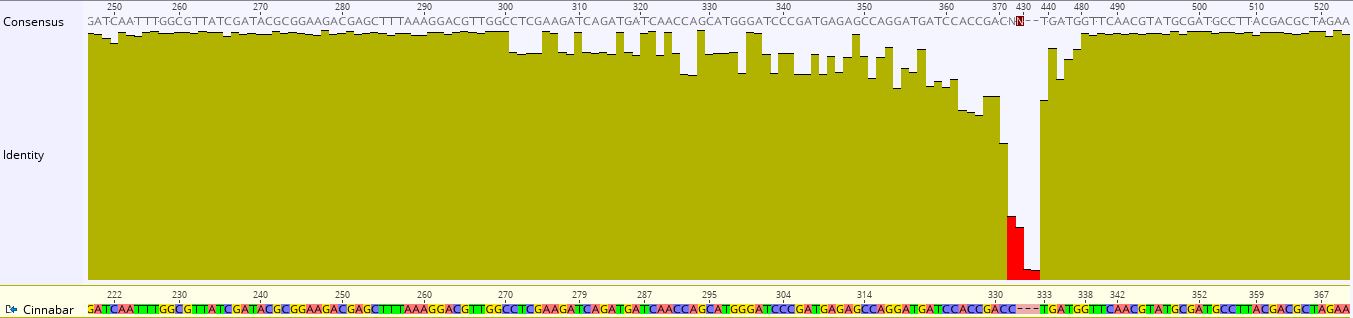

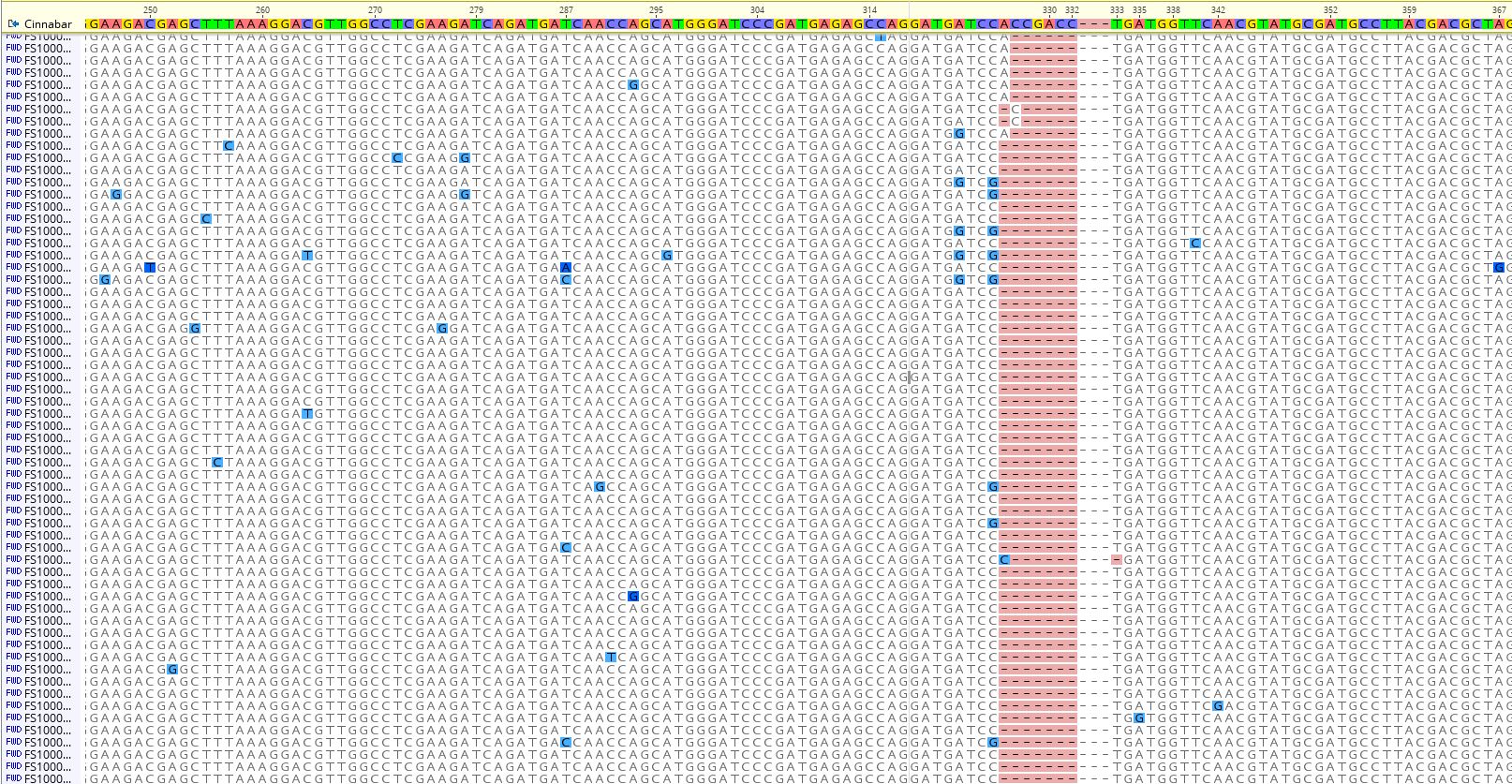


**Figure S22. Mean pairwise identity of all *cinnabar* wing CRISPR (C3) tissue reads against reference (greenish-brown denotes 30-99% identity, red denotes <30% identity). Indels showing deletions at the target site.**


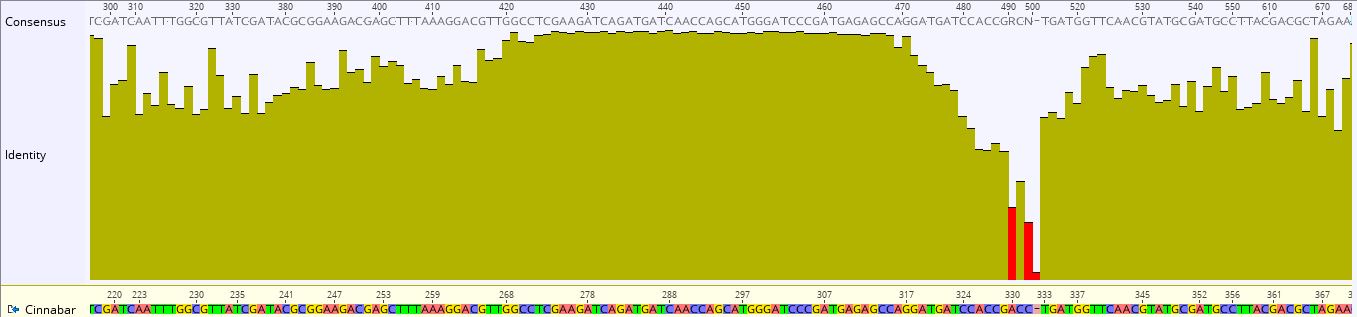

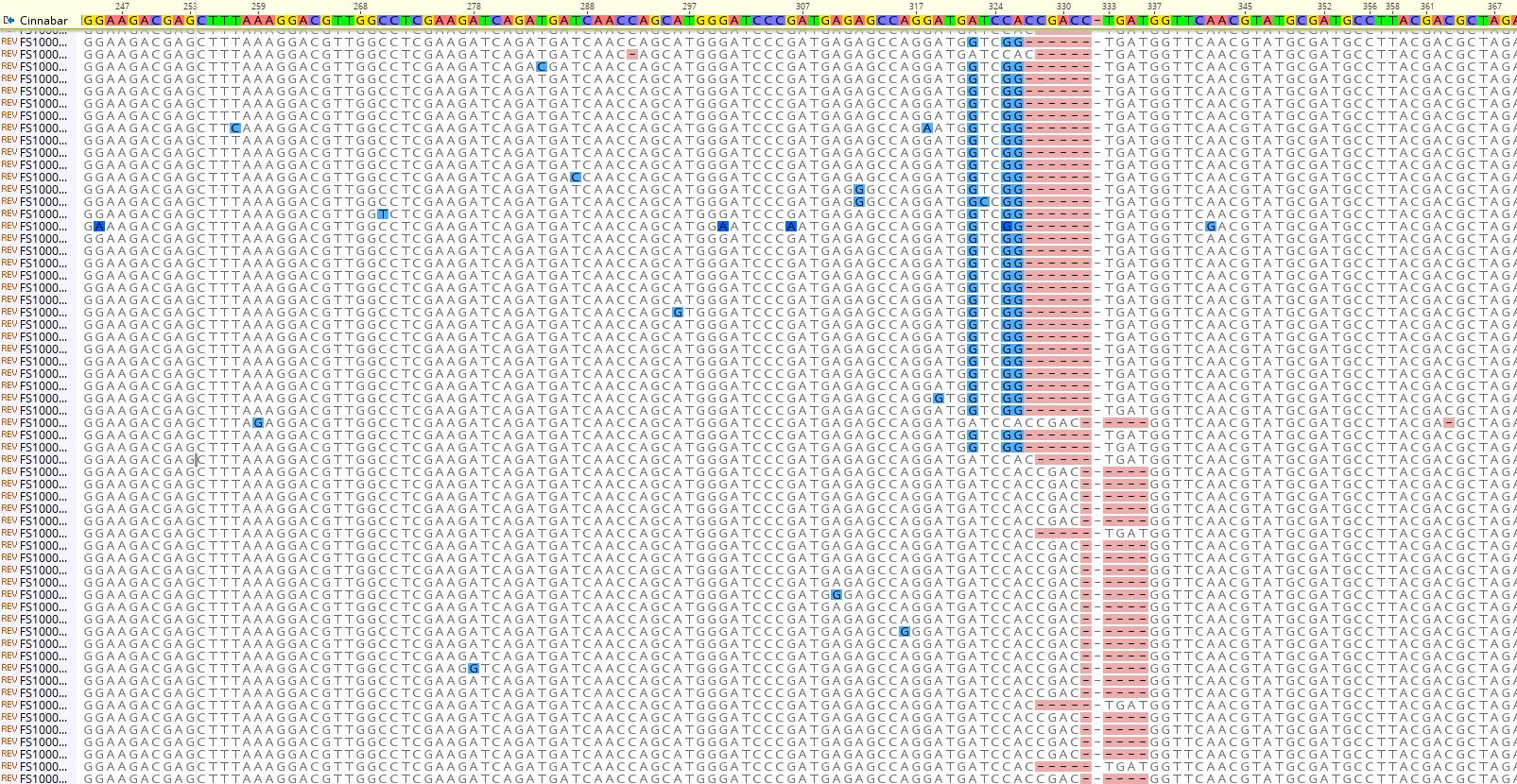


**Figure S23. Mean pairwise identity of all *cinnabar* wing CRISPR (C4) tissue reads against reference (greenish-brown denotes 30-99% identity, red denotes <30% identity). Indels showing deletions at the target site.
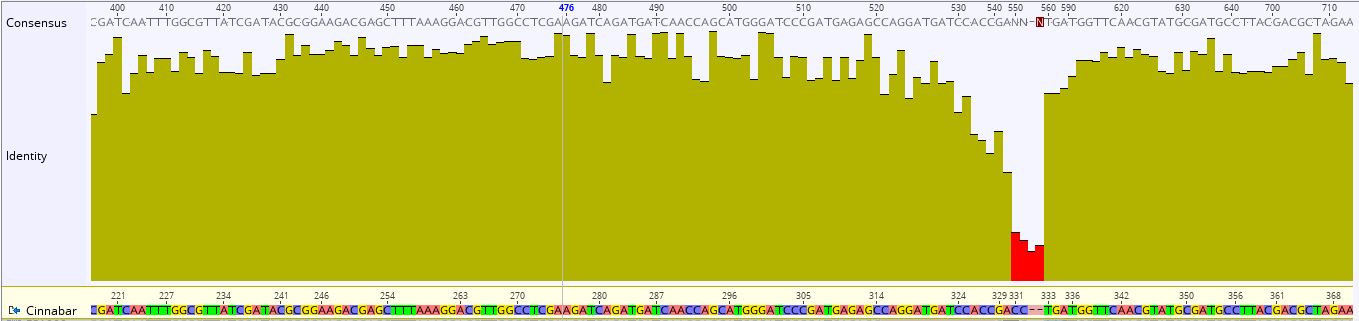
**
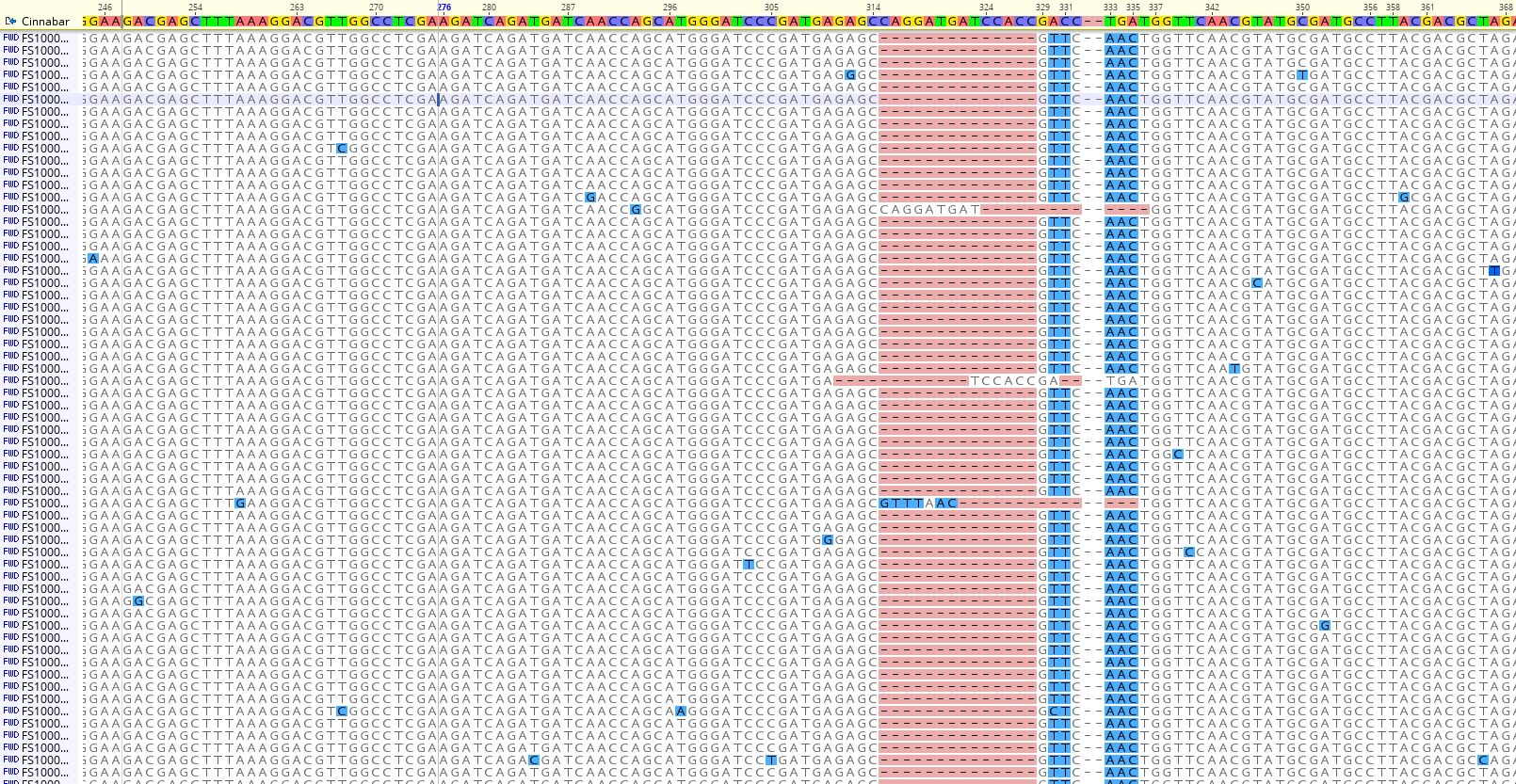


**Figure S24. Mean pairwise identity of all *cinnabar* wing CRISPR (C15) tissue reads against reference (greenish-brown denotes 30-99% identity, red denotes <30% identity). Indels showing deletions at the target site.**


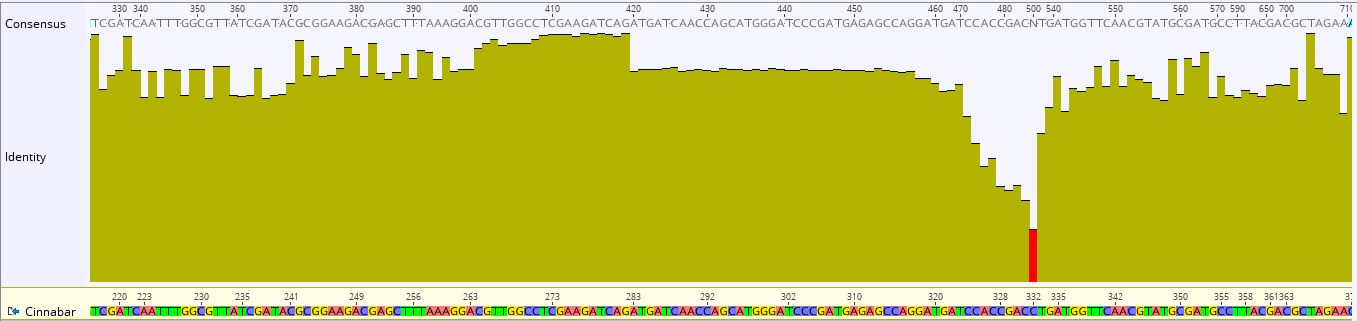

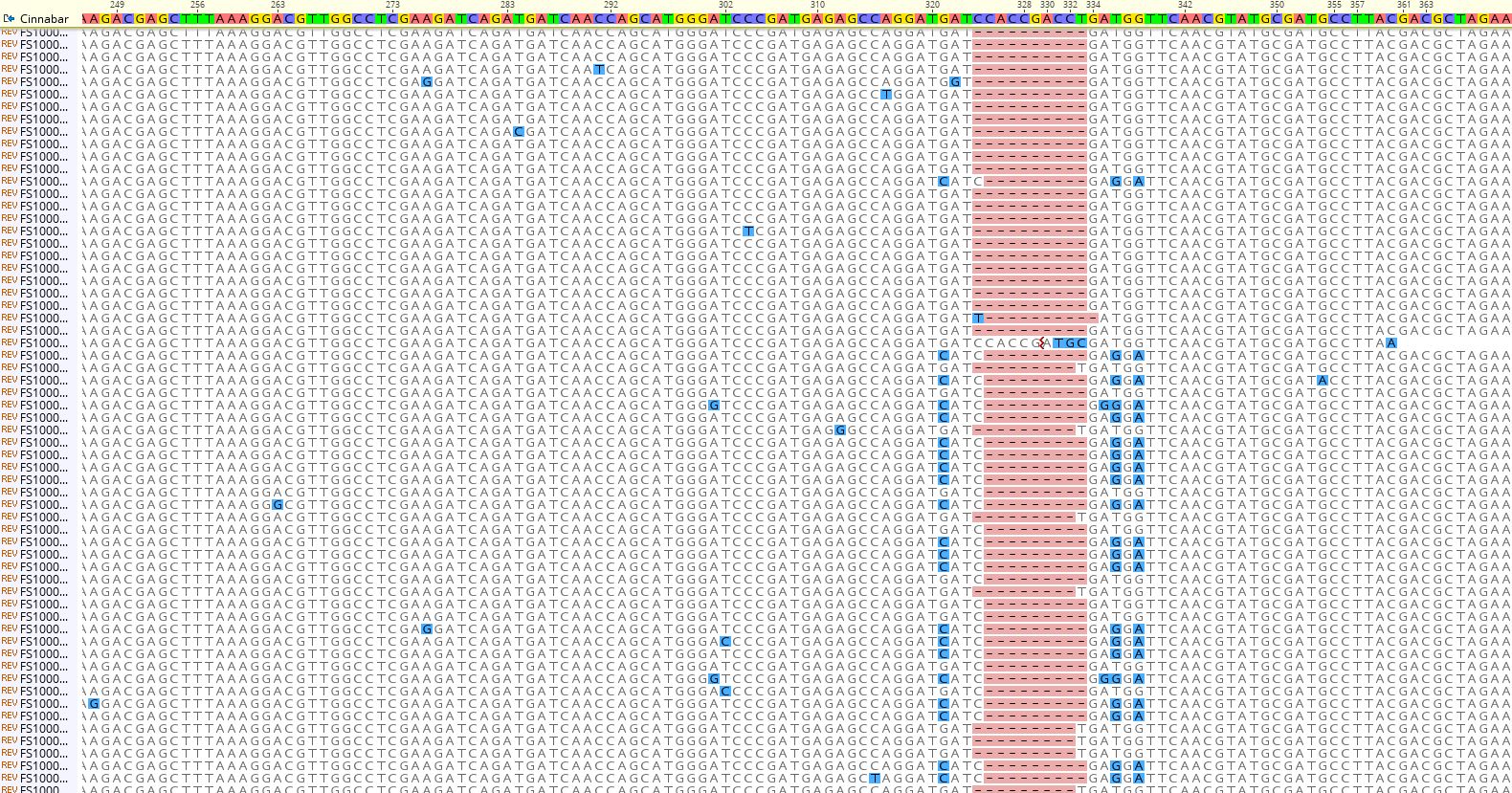


**Figure S25. Mean pairwise identity of all *cinnabar* wing CRISPR (C6) tissue reads against reference (greenish-brown denotes 30-99% identity, red denotes <30% identity). Indels showing deletions at the target site.**


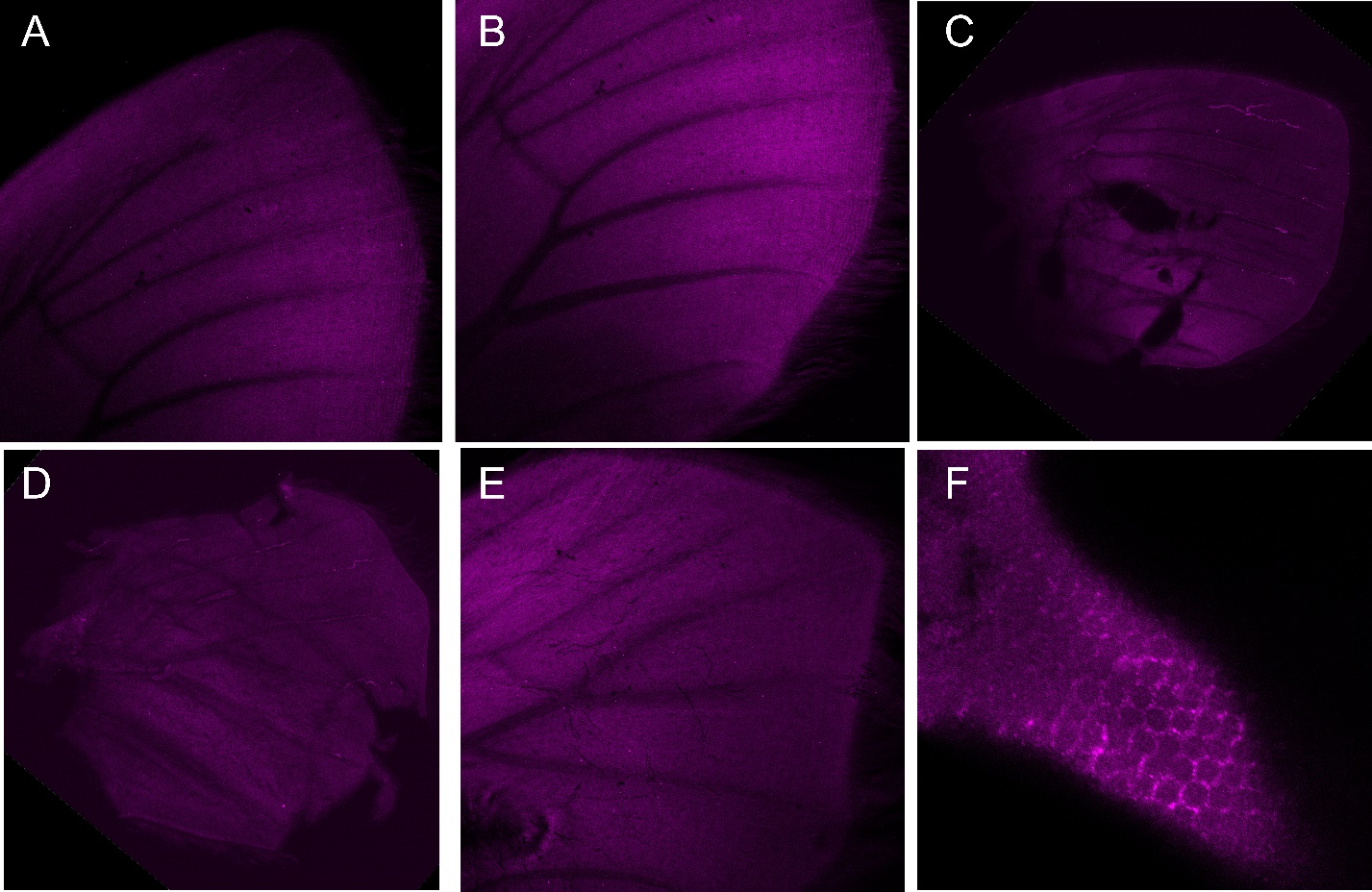


**Figure S26. Expression of *kfase* in pupal wings at 77 to 92% (120-144 hrs) PD and in pupal eyes at 31% PD (48 hrs) in *Bicyclus anynana*.** Expression of *kfase* in the forewings **(A and B),** hindwings (**C-E**), and in the ommatidia (**F**).


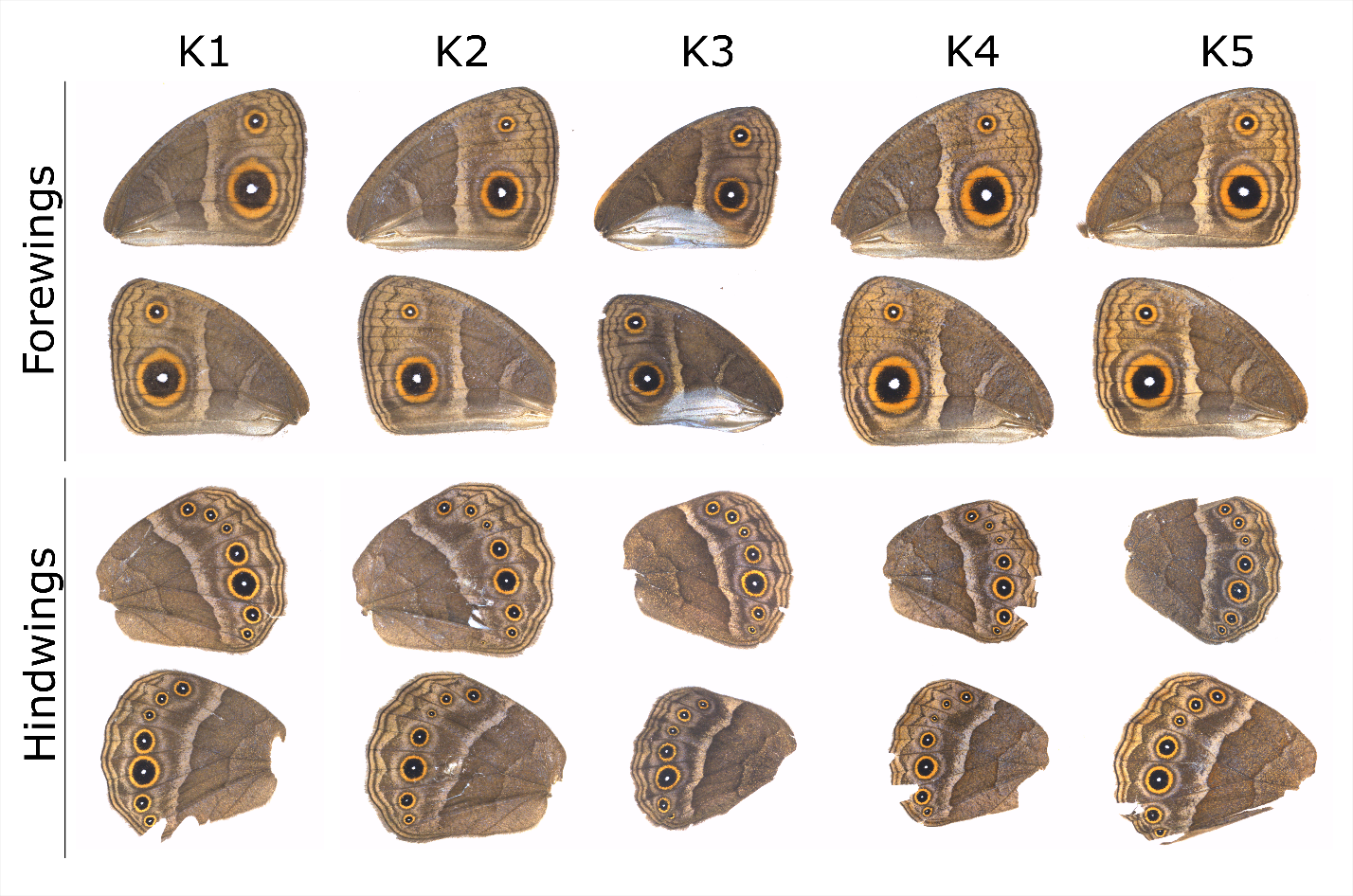


**Figure S27. CRISPR-cas9 on *kfase* in the wings of *Bicyclus anynana*.** No phenotypic defects were observed on wings. Note these wings were not verified by illumina sequencing.


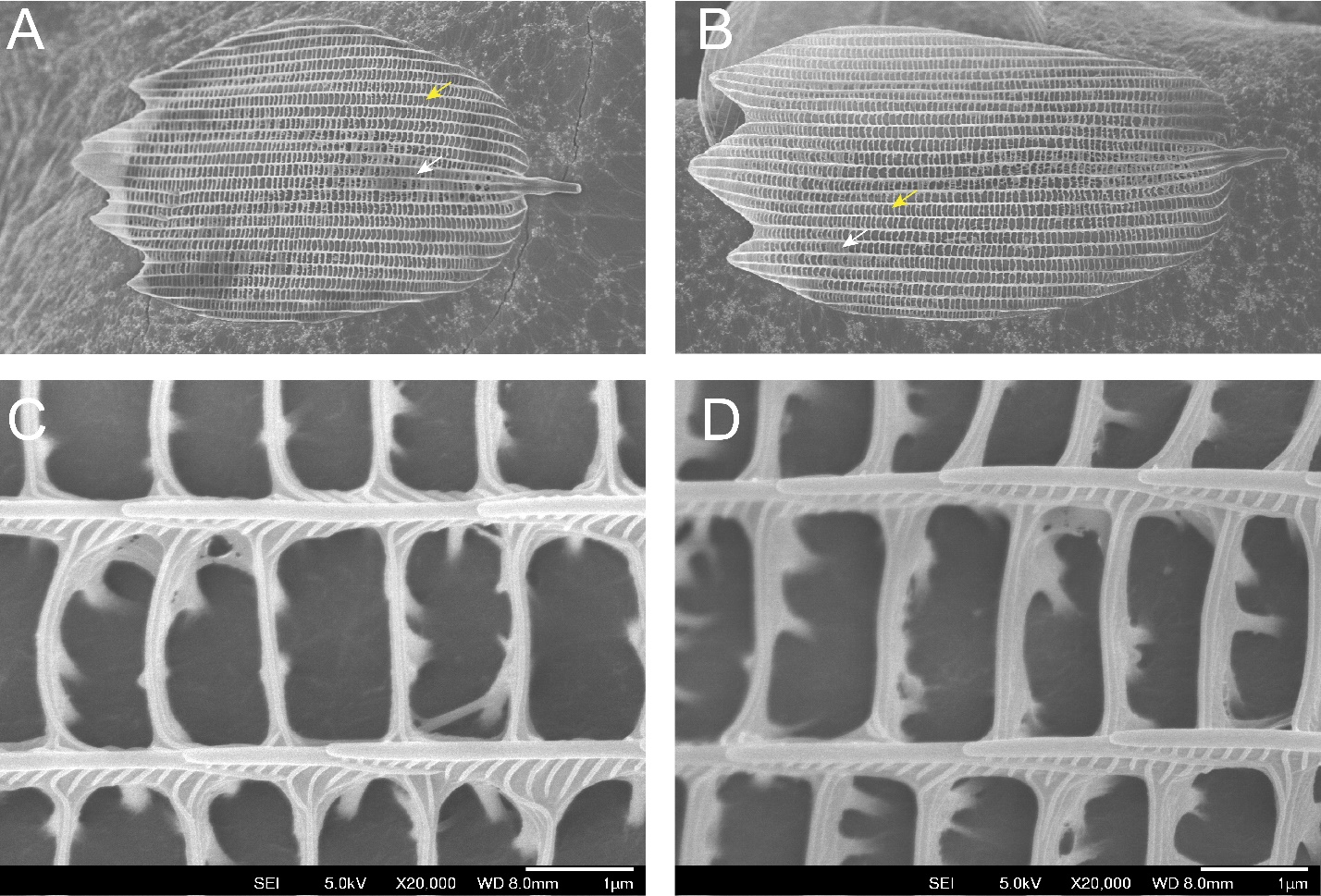


**Figure S28. SEM images of orange scales from the eyespot of *B. anynana*.** No clear pigment granules were observed in the orange scales. Note that some regions of the scales have partially closed windows (white arrow) between crossribs while other have open windows (yellow arrow).

**Table S1: Calculated and theoretical values of retention factor (Rf) for ommochrome pigments using imageJ.**

| Species | Compound | Solvent length | Compound length | Rf | Theoretical Rf |
| --- | --- | --- | --- | --- | --- |
| *B. anynana* | Bromophenol blue | 2706 | 1176 | 0.43 | 0.43 |
| *B. anynana* | Amaranth | 2706 | 354 | 0.13 | 0.13 |
| *B. anynana* | 3-Hydroxy-kynurenine | 2664 | 0 | 0 | 0.56 |
| *B. anynana* | Xanthommatin | 2664 | 0 | 0 | 0.36 |
| *B. anynana* | Ommatin-D | 2664 | 0 | 0 | 0.29 |
| *B. anynana* | Rhodommatin | 2664 | 0 | 0 | 0.23 |
| *B. anynana* | Dihydro-xanthommatin | 2664 | 342 | 0.13 | 0.13 |
|  |  |  |  |  |  |
| *J. almana* | Bromophenol blue | 2592 | 1134 | 0.44 | 0.43 |
| *J. almana* | Amaranth | 2592 | 372 | 0.14 | 0.13 |
| *J. almana* | 3-Hydroxy-kynurenine | 2568 | 0 | 0 | 0.56 |
| *J. almana* | Xanthommatin | 2568 | 0 | 0 | 0.36 |
| *J. almana* | Ommatin-D | 2568 | 714 | 0.28 | 0.29 |
| *J. almana* | Rhodommatin | 2568 | 0 | 0 | 0.23 |
| *J. almana* | Dihydro-xanthommatin | 2568 | 0 | 0 | 0.13 |
|  |  |  |  |  |  |
| *J. orythia* | Bromophenol blue | 2792 | 1232 | 0.44 | 0.43 |
| *J. orythia* | Amaranth | 2792 | 399 | 0.14 | 0.13 |
| *J. orythia* | 3-Hydroxy-kynurenine | 2754 | 0 | 0 | 0.56 |
| *J. orythia* | Xanthommatin | 2754 | 924 | 0.34 | 0.36 |
| *J. orythia* | Ommatin-D | 2754 | 732 | 0.27 | 0.29 |
| *J. orythia* | Rhodommatin | 2754 | 0 | 0 | 0.23 |
| *J. orythia* | Dihydro-xanthommatin | 2754 | 0 | 0 | 0.13 |

**Table S2: Microinjection table with number of embryos injected with gRNA targeting each gene, and their outcome.**

| **Target** | **Injected** | **Hatched** | **Mosaic (eyes)** |
| --- | --- | --- | --- |
| *vermilion* | 306 | 58 | 5 |
| *cinnabar* | 378 | 53 | 6 |
| *kynurenine formamidase* | 381 | 73 | 0 |

**Table S3: CRISPR-Cas9 RNA guides**

| **Target** | **Sequence (5’-3’)** |
| --- | --- |
| *optix* | GCACCCCAACGTGGCCGAGC |
| *cinnabar* | AGGATGATCCACCGACCTGA |
| *vermilion* | CCTGGAAACAATGACTCCAC |
| *kynurenine formamidase* | TTGTAGTGGGCTACGACCTT |

**Table S4: Primers for next-generation sequencing and cDNA PCR amplification**

| **Target** | **Sequence** | |
| --- | --- | --- |
| *cinnabar* | F | CGATCAATTTGGCGTTATC |
|  | R | TTCTAGCGTCGTAAGGCATC |
| *vermilion* | F | CTTCTAGTGGACCAGGTGATG |
|  | R | CTTATTCTCCAAAAGTCG |
| *kynurenine formamidase* | F | CACCCGTCTTAGTGTACATCC |
|  | R | CAGCGTTCTGTATTTGGTTG |
| *ef1α* | F | GTTGAGATGCACCACGAAGC |
|  | R | CATAGCCACGACGCAATTCC |

**Table S5: Transcript per million values of *vermilion, cinnabar*, and *kfase* in the 24 hrs wing disc of *B. anynana* (Data from Murugesan et al., 2022).**

| **Genes** | **Pupal 24hr forewing** | | | |
| --- | --- | --- | --- | --- |
|  | **Bio replicate 1** | **Bio replicate 2** | **Bio replicate 3** | **Bio replicate 4** |
| Bany_03205/ *cinnabar* | 2.521949 | 2.417296 | 2.727554 | 2.108283 |
| Bany_16589/ *vermilion* | 22.56011 | 22.09697 | 20.27641 | 20.63847 |
| Bany_00328/ *kynurenine formamidase* | 38.01949 | 33.73774 | 28.62013 | 35.85073 |

|  |
| --- |
|  |
|  |

**HCR Probes**

For each gene sequence, binding sites for split-initiator probes P1 and P2 are highlighted in green and yellow, respectively. Both P1 and P2, each carrying one half of the HCR initiator I1 binding site are required for subsequent binding of HCR amplifiers. Full P1 and P2 probe sequences are included in tables below each sequence. Spacer bases are highlighted in blue.

>Vermilion (tryptophan 2,3-dioxygenase)_XM_024097096.2_

Link: https://www.ncbi.nlm.nih.gov/nuccore/XM_024097096.2/

ATGAGGTCGGTGACAGATGACTCGGCCCAAGATGGTGACCACCTGGGCAACGAGGCTGGTATGCTCTATGGCGAATACCTGATGCTGGACAAATTGCTCAGTGCTCAGAGGATGCTCAGCGCTGAGTCTTCCAAACCTGTTCATGACGAGCATCTGTTTATAGTGACACATCAAGCATACGAGCTATGGTTCAAGCAGATAATCTTCGAAGTGGACTCGGTGAGGACTCTGCTGGACGTGGAAGGTCTAGATGAGAGTCACACCATGGAAATCTTGAAGAGGCTAAACAGAGTTGTACTAATTCTCAAGxxCTTCTAGTGGACCAGGTGATGATCCTGGAAACAATGACTCCACTGGACTTCATGGACTTCAGGCACTATCTGCGACCGGCGTCCGGGTTCCAGAGTTTACAGTTCCGACTTTTGGAGAATAAGCTCGGTTTAAAACAAGCTCTACGGGTGAAATACAACCAAAACTATCAAACTGTATTCGGTGACGATCCAGAAGCTATGGACTCTTTACATAAATCTGAAGAAGAGCCAGCGCTACTAGCCCTAATAGAGCGGTGGTTGGAACGCACGCCGGGCCTCATTACACACGGGTTCGATTTTTGGGGCAAATTCCAGGCTGCAGTTGACAAAATGATCAGGGAATCTGTTGAAGAAGCTATGCGTGAACCAAACGAAACGGTTCGTCGCCACCGTCTGCAAGACGCGGAGAACCGACGCGAGATCTACCGCTCCATCTTCGATCCCGCCGTGCACGACGCGCTGCGATCCCGCGGCGAGCGGCGACTGTCGCACCGCGCACTGCAAGGCGCCATCATGATAACGTTCTACCGCGACGAGCCGCGCTTCTCACAGCCGCACCAGCTGCTCACGCTGCTGATGGACATGGACAGCCTCATCACCAAGTGGCGCTATAACCACGTGATAATGGTACAACGCATGATCGGCTCGCAGCAGCTCGGTACAGGCGGCTCTTCGGGCTACCAGTACCTGCGCTCCACACTCAGTGACCGATATAAAGTGTTCCTGGACTTGTTCAACTTATCTACGTTCCTTCTGCCGCGTTCCCTCATCCCGCCCCTGGACGACGGGATAAAGAGGGACCTCAGCCTGACCTGGGGAGATCACATCAGGGAAAATGGACACAGCGCCAAAGAAAACGGGGAAAATAAGAAAAATGGGGAAAACCACCAAAACGGTTTGGATGAATTTTCAAAACTTAGTTTGAATGAAAAGAAATCATAA

| **Name** | **Sequence** |
| --- | --- |
| vrm1_HCR_P1B1 | gAggAgggCAgCAAACggAAATCTTGGGCCGAGTCATCTGTCACC |
| vrm1_HCR_P2B1 | ACCAGCCTCGTTGCCCAGGTGGTCATAgAAgAgTCTTCCTTTACg |
| vrm2_HCR_P1B1 | gAggAgggCAgCAAACggAAGAGCAATTTGTCCAGCATCAGGTAT |
| vrm2_HCR_P2B1 | CTCAGCGCTGAGCATCCTCTGAGCATAgAAgAgTCTTCCTTTACg |
| vrm3_HCR_P1B1 | gAggAgggCAgCAAACggAAAGCAGAGTCCTCACCGAGTCCACTT |
| vrm3_HCR_P2B1 | TGACTCTCATCTAGACCTTCCACGTTAgAAgAgTCTTCCTTTACg |
| vrm4_HCR_P1B1 | gAggAgggCAgCAAACggAAGTACAACTCTGTTTAGCCTCTTCAA |
| vrm4_HCR_P2B1 | CCTGGTCCACTAGAAGCTTGAGAATTAgAAgAgTCTTCCTTTACg |
| vrm5_HCR_P1B1 | gAggAgggCAgCAAACggAACTTATTCTCCAAAAGTCGGAACTGT |
| vrm5_HCR_P2B1 | CACCCGTAGAGCTTGTTTTAAACCGTAgAAgAgTCTTCCTTTACg |
| vrm6_HCR_P1B1 | gAggAgggCAgCAAACggAAATCGTCACCGAATACAGTTTGATAG |
| vrm6_HCR_P2B1 | TTTATGTAAAGAGTCCATAGCTTCTTAgAAgAgTCTTCCTTTACg |
| vrm7_HCR_P1B1 | gAggAgggCAgCAAACggAAACTGCAGCCTGGAATTTGCCCCAAA |
| vrm7_HCR_P2B1 | TCAACAGATTCCCTGATCATTTTGTTAgAAgAgTCTTCCTTTACg |
| vrm8_HCR_P1B1 | gAggAgggCAgCAAACggAATGGCGACGAACCGTTTCGTTTGGTT |
| vrm8_HCR_P2B1 | CGTCGGTTCTCCGCGTCTTGCAGACTAgAAgAgTCTTCCTTTACg |

>Cinnabar (kynurenine 3-monooxygenase-like)_XM_024096156.1

Link: https://www.ncbi.nlm.nih.gov/nuccore/XM_024096156.1

ATGCAAAAAAGTACATTTGAGAATTGTAATCCTAAGAATCTCACAGACGAGGTGTTGAAATATAACGGAAATGCAAATAAGAAATTGAATATCGCTGTCGTTGGCGGAGGACTTGTGGGATCCCTTGAAGCATTGTACTTAGCCAAAAGAGGACATCAAGTCAAGCTTTATGAGTATCGTGAAGATATAAGAAATACACCGTTAGCTCGAGGAAGGTCGATCAATTTGGCGTTATCGATACGCGGAAGACGAGCTTTAAAGGACGTTGGCCTCGAAGATCAGATGATCAACCAGCATGGGATCCCGATGAGAGCCAGGATGATCCACCGACCTGATGGTTCAACGTATGCGATGCCTTACGACGCTAGAACTAACCAGTGCATATATTCCGTGGGGAGAAATTATCTAAACAGCATTCTACTGAAAGAATCGGAAAACTTGGACAATGTTGAGAGATACTTTAATCACAAGTTGATCAAATCAAACTTTAAGGATGGATCGTTAACATTTATGAAGATGGACACAAAAGAGACGATTCAAGTGAACGCAGACTTGATAATCGGCGCGGATGGTGCATTTTCAACAGTTCGTAAGGAAATGATGAAGCAACCACTATTTAACTTCAGCCAGCAGTACATAGAGCATGGCTATTTGGAGCTCTGTATACCTGCTGGTAAAGATGGTGGTTTTCAGATGCCATCAAACTATTTACACATTTGGCCAAGAGGAAACTTCATGATGATCGCACTGCCGAATCAGGATTGTTCATGGACAGTTACACTGTTTATGCCATTTAAAAACTTCAAGAACATTGATTCTGAAAGAAAGCTTATGGACTTCTTTACAAACTACTTTCAAGATGCCATACCACTCATAGGAGAAAAGAAGTTGATTGAAGATTTTTTTAGTGGATCACCTTCAGCGTTGGTGGCAATTAAGTGCCGTCCCTATCACGTCTGTGATAAAGCTCTTCTGATTGGTGATGCAGCACATGCGGTCGTGCCTTTCTACGGCCAAGGGATGAACGCCGGCTTTGAAGACTGTACTGAACTAGACAAACTCTTCCAGAAGTATAACGATGACGTCACAAGCGTACTCGAAGAGTTTACTAACACCAGATGGCGTGATGCTTTTGCGATCAGCGACTTGGCTATGTACAATTATATAGAGATGCGCGACCTTGTTACTCGTCCATCGTATCGGCTTCGAAAAACCGTCGATGATTTCTTATATTGGCTGCTACCCGATTTATGGGTACCTCTTTACAATTCTGTTACTTTTTCAACAATGCCTTATGATCAGTGTGTAAAAAACCGCCAATGGCAAAATAAGGTCCTTGCCATCGTTGTCCTGTTTTTTGGATTGATGCTATTTGTAACCATCTATAGAAATATGTTGTTATAG

| **Name** | **Sequence** |
| --- | --- |
| cin1_HCR_P1B1 | gAggAgggCAgCAAACggAACGACAGCGATATTCAATTTCTTATT |
| cin1_HCR_P2B1 | CAAGGGATCCCACAAGTCCTCCGCCTAgAAgAgTCTTCCTTTACg |
| cin2_HCR_P1B1 | gAggAgggCAgCAAACggAAGCTTGACTTGATGTCCTCTTTTGGC |
| cin2_HCR_P2B1 | TTCTTATATCTTCACGATACTCATATAgAAgAgTCTTCCTTTACg |
| cin3_HCR_P1B1 | gAggAgggCAgCAAACggAACATGCTGGTTGATCATCTGATCTTC |
| cin3_HCR_P2B1 | GGATCATCCTGGCTCTCATCGGGATTAgAAgAgTCTTCCTTTACg |
| cin4_HCR_P1B1 | gAggAgggCAgCAAACggAAGCGTCGTAAGGCATCGCATACGTTG |
| cin4_HCR_P2B1 | ACGGAATATATGCACTGGTTAGTTCTAgAAgAgTCTTCCTTTACg |
| cin5_HCR_P1B1 | gAggAgggCAgCAAACggAACCATCCTTAAAGTTTGATTTGATCA |
| cin5_HCR_P2B1 | GTGTCCATCTTCATAAATGTTAACGTAgAAgAgTCTTCCTTTACg |
| cin6_HCR_P1B1 | gAggAgggCAgCAAACggAAGCCGATTATCAAGTCTGCGTTCACT |
| cin6_HCR_P2B1 | ACGAACTGTTGAAAATGCACCATCCTAgAAgAgTCTTCCTTTACg |
| cin7_HCR_P1B1 | gAggAgggCAgCAAACggAAGCATCTGAAAACCACCATCTTTACC |
| cin7_HCR_P2B1 | TTGGCCAAATGTGTAAATAGTTTGATAgAAgAgTCTTCCTTTACg |
| cin8_HCR_P1B1 | gAggAgggCAgCAAACggAACAATCCTGATTCGGCAGTGCGATCA |
| cin8_HCR_P2B1 | GGCATAAACAGTGTAACTGTCCATGTAgAAgAgTCTTCCTTTACg |

> kynurenine formamidase_XM_024095364.1

Link: https://www.ncbi.nlm.nih.gov/nuccore/XM_024095364.1

ATGGCGCTGATAACCGAAGGGATCGATCTAGAGAGGGAGTATTCGCCCAGCATGTGGTCGAAGAGGTTCTCTAGTCCCCAAGAAGTTACGCAGCATCATGTAAACGTCGTCACTGCAGCAAGCGAAGCAGTAATCAACACCTTGCCTCATAAACTAGAGATAGAATATGGTTGTACTCCGGGACAGAAGTTGGACATAATAGGCACAGACCTCCCTGATGATTCACCCGTCTTAGTGTACATCCACGGCGGCTATTGGCAGATGCTGTCGCGGGAAGCATCGCGGTACCTCGCCCAGCCGCTGCACCGGTTCCGCATCAAGACCATTGTAGTGGGCTACGACCTTTGTCCAGTTGCCACGCTGCCGGAAATAGTCAACCAAATACAGAACGCTGCCAAATTCGTATTCGAGTACGCAGAGAAGATGGGCTCGAGAGGCGTCTACTTCGCCGGCCACTCAGCGGGAGCTCATCTAGTAGCAAAGTTGCTGTCCAATTCAGATTTCCTCGACAGCACAGAAGGTTCCCACCGTCTGCAAGGTGCCTTCCTGATCTCAGGAATCTTCGACTTAAGAGAAGTCATCCATACATCTGTGAACCTTGCAGTGCAGTTGCCCGTCGAATGGGCAGTGCCTCTATCTCCTCAGTTTGACTGCTTCACGCATCTGCAAGCAAGACGAGTGCGTGTATACATTTTAGCGGGACAGTATGATAGCCCGACGTTCAAGAAGCAATCGAGAGAGTTCTACGAACTCTTGCACAATACCTGTTTGATGCAAAACATGTACTTGGAAATCAAAGATAATTTTGACCATTTTGACATCGTGGAGTGTTTTGCCCAAGATGACAATTACCTTAGGAAATTATTGGTGCACGATATTCGTAAGCATCTTTAA

| **Name** | **Sequence** |
| --- | --- |
| kfase1_HCR_P1B1 | gAggAgggCAgCAAACggAACTCTAGATCGATCCCTTCGGTTATC |
| kfase1_HCR_P2B1 | CGACCACATGCTGGGCGAATACTCCTAgAAgAgTCTTCCTTTACg |
| kfase2_HCR_P1B1 | gAggAgggCAgCAAACggAAATGATGCTGCGTAACTTCTTGGGGA |
| kfase2_HCR_P2B1 | TTCGCTTGCTGCAGTGACGACGTTTTAgAAgAgTCTTCCTTTACg |
| kfase3_HCR_P1B1 | gAggAgggCAgCAAACggAAGACGGGTGAATCATCAGGGAGGTCT |
| kfase3_HCR_P2B1 | CCAATAGCCGCCGTGGATGTACACTTAgAAgAgTCTTCCTTTACg |
| kfase4_HCR_P1B1 | gAggAgggCAgCAAACggAACTGGGCGAGGTACCGCGATGCTTCC |
| kfase4_HCR_P2B1 | GGTCTTGATGCGGAACCGGTGCAGCTAgAAgAgTCTTCCTTTACg |
| kfase5_HCR_P1B1 | gAggAgggCAgCAAACggAAAGCCCATCTTCTCTGCGTACTCGAA |
| kfase5_HCR_P2B1 | AGTGGCCGGCGAAGTAGACGCCTCTTAgAAgAgTCTTCCTTTACg |
| kfase6_HCR_P1B1 | gAggAgggCAgCAAACggAAGAATTGGACAGCAACTTTGCTACTA |
| kfase6_HCR_P2B1 | GAACCTTCTGTGCTGTCGAGGAAATTAgAAgAgTCTTCCTTTACg |
| kfase7_HCR_P1B1 | gAggAgggCAgCAAACggAAGGCACTGCCCATTCGACGGGCAACT |
| kfase7_HCR_P2B1 | GTGAAGCAGTCAAACTGAGGAGATATAgAAgAgTCTTCCTTTACg |
| kfase8_HCR_P1B1 | gAggAgggCAgCAAACggAACCGCTAAAATGTATACACGCACTCG |
| kfase8_HCR_P2B1 | TCTTGAACGTCGGGCTATCATACTGTAgAAgAgTCTTCCTTTACg |

>Optix_XM_024080404.2

Link: https://www.ncbi.nlm.nih.gov/nuccore/XM_024080404.2

ATGCGCGGCTCCTGGGACGAGTCCACGACGGCGGCGCTGCACGCGCGCATCCTGGAGGCGCACCGCGGGTCCGCCGCGCCCGACCGCGCCGAGCCCGCGTGCGAGCCTCCGCCGCTGACGCTGGGCGCGCTGGAGCTGGCGGCGCCCACGCCGCTGCTGCCGCTGCCCACGCTGAGCTTCAGCGCCGCGCAGGTGGCCACCGTGTGCGAGACGCTGGAGGAGAGCGGCGACGTGGAGCGCCTGGCGCGCTTCTTGTGGTCGCTGCCCGTGGCGCACCCCAACGTGGCCGAGCTGGAGCGCTGCGAAGCCGTGCTGCGCGCGCGCGCCGTCGTCGCCTTCCACGCCGGCCGCCACCGCGAGCTGTACGCCATCCTCGAGCGCCACCGCTTCCAGCGCTCCAGCCACGCCAAGCTGCAAGCGCTGTGGCTGGAGGCGCACTACCAGGAGGCTGAGCGCCTGCGCGGCCGTCCGCTGGGCCCCGTCGACAAGTACCGCGTGCGGAAGAAGTTCCCGCTCCCGAGGACGATCTGGGACGGCGAGCAGAAGACGCACTGTTTCAAGGAGCGGACGCGATCTCTACTCCGAGAATGGTACCTCCAAGATCCCTACCCGAACCCGACGAAGAAGAGGGAATTGGCGGCGGCGACGGGTCTGACGCCGACGCAAGTCGGCAACTGGTTCAAAAACCGACGGCAAAGAGACCGAGCGGCCGCCGCCAAGAACCGCTCCGCCGTGCTGGGCAGAGGATAA

| **Name** | **Sequence** |
| --- | --- |
| Optix1_HCR_P1B2 | CCTCgTAAATCCTCATCAAACGCCGTCGTGGACTCGTCCCAG |
| Optix1_HCR_P2B2 | CGCCTCCAGGATGCGCGCGTGCAGCAAATCATCCAgTAAACCgCC |
| Optix2_HCR_P1B2 | CCTCgTAAATCCTCATCAAAGGCTCGGCGCGGTCGGGCGCGGCGG |
| Optix2_HCR_P2B2 | AGCGTCAGCGGCGGAGGCTCGCACGAAATCATCCAgTAAACCgCC |
| Optix3_HCR_P1B2 | CCTCgTAAATCCTCATCAAAGGCAGCAGCGGCGTGGGCGCCGCCA |
| Optix3_HCR_P2B2 | GCGGCGCTGAAGCTCAGCGTGGGCAAAATCATCCAgTAAACCgCC |
| Optix4_HCR_P1B2 | CCTCgTAAATCCTCATCAAACCGCTCTCCTCCAGCGTCTCGCACA |
| Optix4_HCR_P2B2 | AAGAAGCGCGCCAGGCGCTCCACGTAAATCATCCAgTAAACCgCC |
| Optix5_HCR_P1B2 | CCTCgTAAATCCTCATCAAAGCGGTGGCGGCCGGCGTGGAAGGCG |
| Optix5_HCR_P2B2 | GTGGCGCTCGAGGATGGCGTACAGCAAATCATCCAgTAAACCgCC |
| Optix6_HCR_P1B2 | CCTCgTAAATCCTCATCAAAACAGCGCTTGCAGCTTGGCGTGGCT |
| Optix6_HCR_P2B2 | CAGCCTCCTGGTAGTGCGCCTCCAGAAATCATCCAgTAAACCgCC |
| Optix7_HCR_P1B2 | CCTCgTAAATCCTCATCAAAACAGTGCGTCTTCTGCTCGCCGTCC |
| Optix7_HCR_P2B2 | GAGTAGAGATCGCGTCCGCTCCTTGAAATCATCCAgTAAACCgCC |
| Optix8_HCR_P1B2 | CCTCgTAAATCCTCATCAAACGTCGGGTTCGGGTAGGGATCTTGG |
| Optix8_HCR_P2B2 | CGTCGCCGCCGCCAATTCCCTCTTCAAATCATCCAgTAAACCgCC |
| Optix9_HCR_P1B2 | CCTCgTAAATCCTCATCAAATTTGAACCAGTTGCCGACTTGCGTC |
| Optix9_HCR_P2B2 | GGCCGCTCGGTCTCTTTGCCGTCGGAAATCATCCAgTAAACCgCC |

**CRISPR-Cas9 deletion sites**

crRNA sequences (Supplementary Table 3) are highlighted in red.

**>**Vermilion_ XM_024097096.2

Link: https://www.ncbi.nlm.nih.gov/nuccore/XM_024097096.2/

ATGGCGTGTCCTATGAGGTCGGTGACAGATGACTCGGCCCAAGATGGTGACCACCTGGGCAACGAGGCTGGTATGCTCTATGGCGAATACCTGATGCTGGACAAATTGCTCAGTGCTCAGAGGATGCTCAGCGCTGAGTCTTCCAAACCTGTTCATGACGAGCATCTGTTTATAGTGACACATCAAGCATACGAGCTATGGTTCAAGCAGATAATCTTCGAAGTGGACTCGGTGAGGACTCTGCTGGACGTGGAAGGTCTAGATGAGAGTCACACCATGGAAATCTTGAAGAGGCTAAACAGAGTTGTACTAATTCTCAAGCTTCTAGTGGACCAGGTGATGATCCTGGAAACAATGACTCCACTGGACTTCATGGACTTCAGGCACTATCTGCGACCGGCGTCCGGGTTCCAGAGTTTACAGTTCCGACTTTTGGAGAATAAGCTCGGTTTAAAACAAGCTCTACGGGTGAAATACAACCAAAACTATCAAACTGTATTCGGTGACGATCCAGAAGCTATGGACTCTTTACATAAATCTGAAGAAGAGCCAGCGCTACTAGCCCTAATAGAGCGGTGGTTGGAACGCACGCCGGGCCTCATTACACACGGGTTCGATTTTTGGGGCAAATTCCAGGCTGCAGTTGACAAAATGATCAGGGAATCTGTTGAAGAAGCTATGCGTGAACCAAACGAAACGGTTCGTCGCCACCGTCTGCAAGACGCGGAGAACCGACGCGAGATCTACCGCTCCATCTTCGATCCCGCCGTGCACGACGCGCTGCGATCCCGCGGCGAGCGGCGACTGTCGCACCGCGCACTGCAAGGCGCCATCATGATAACGTTCTACCGCGACGAGCCGCGCTTCTCACAGCCGCACCAGCTGCTCACGCTGCTGATGGACATGGACAGCCTCATCACCAAGTGGCGCTATAACCACGTGATAATGGTACAACGCATGATCGGCTCGCAGCAGCTCGGTACAGGCGGCTCTTCGGGCTACCAGTACCTGCGCTCCACACTCAGTGACCGATATAAAGTGTTCCTGGACTTGTTCAACTTATCTACGTTCCTTCTGCCGCGTTCCCTCATCCCGCCCCTGGACGACGGGATAAAGAGGGACCTCAGCCTGACCTGGGGAGATCACATCAGGGAAAATGGACACAGCGCCAAAGAAAACGGGGAAAATAAGAAAAATGGGGAAAACCACCAAAACGGTTTGGATGAATTTTCAAAACTTAGTTTGAATGAAAAGAAATCATAA

>Cinnabar_XM_024096156.1

Link: https://www.ncbi.nlm.nih.gov/nuccore/XM_024096156.1

ATGCAAAAAAGTACATTTGAGAATTGTAATCCTAAGAATCTCACAGACGAGGTGTTGAAATATAACGGAAATGCAAATAAGAAATTGAATATCGCTGTCGTTGGCGGAGGACTTGTGGGATCCCTTGAAGCATTGTACTTAGCCAAAAGAGGACATCAAGTCAAGCTTTATGAGTATCGTGAAGATATAAGAAATACACCGTTAGCTCGAGGAAGGTCGATCAATTTGGCGTTATCGATACGCGGAAGACGAGCTTTAAAGGACGTTGGCCTCGAAGATCAGATGATCAACCAGCATGGGATCCCGATGAGAGCCAGGATGATCCACCGACCTGATGGTTCAACGTATGCGATGCCTTACGACGCTAGAACTAACCAGTGCATATATTCCGTGGGGAGAAATTATCTAAACAGCATTCTACTGAAAGAATCGGAAAACTTGGACAATGTTGAGAGATACTTTAATCACAAGTTGATCAAATCAAACTTTAAGGATGGATCGTTAACATTTATGAAGATGGACACAAAAGAGACGATTCAAGTGAACGCAGACTTGATAATCGGCGCGGATGGTGCATTTTCAACAGTTCGTAAGGAAATGATGAAGCAACCACTATTTAACTTCAGCCAGCAGTACATAGAGCATGGCTATTTGGAGCTCTGTATACCTGCTGGTAAAGATGGTGGTTTTCAGATGCCATCAAACTATTTACACATTTGGCCAAGAGGAAACTTCATGATGATCGCACTGCCGAATCAGGATTGTTCATGGACAGTTACACTGTTTATGCCATTTAAAAACTTCAAGAACATTGATTCTGAAAGAAAGCTTATGGACTTCTTTACAAACTACTTTCAAGATGCCATACCACTCATAGGAGAAAAGAAGTTGATTGAAGATTTTTTTAGTGGATCACCTTCAGCGTTGGTGGCAATTAAGTGCCGTCCCTATCACGTCTGTGATAAAGCTCTTCTGATTGGTGATGCAGCACATGCGGTCGTGCCTTTCTACGGCCAAGGGATGAACGCCGGCTTTGAAGACTGTACTGAACTAGACAAACTCTTCCAGAAGTATAACGATGACGTCACAAGCGTACTCGAAGAGTTTACTAACACCAGATGGCGTGATGCTTTTGCGATCAGCGACTTGGCTATGTACAATTATATAGAGATGCGCGACCTTGTTACTCGTCCATCGTATCGGCTTCGAAAAACCGTCGATGATTTCTTATATTGGCTGCTACCCGATTTATGGGTACCTCTTTACAATTCTGTTACTTTTTCAACAATGCCTTATGATCAGTGTGTAAAAAACCGCCAATGGCAAAATAAGGTCCTTGCCATCGTTGTCCTGTTTTTTGGATTGATGCTATTTGTAACCATCTATAGAAATATGTTGTTATAG

> kynurenine formamidase_XM_024095364.1

Link: https://www.ncbi.nlm.nih.gov/nuccore/XM_024095364.1

ATGGCGCTGATAACCGAAGGGATCGATCTAGAGAGGGAGTATTCGCCCAGCATGTGGTCGAAGAGGTTCTCTAGTCCCCAAGAAGTTACGCAGCATCATGTAAACGTCGTCACTGCAGCAAGCGAAGCAGTAATCAACACCTTGCCTCATAAACTAGAGATAGAATATGGTTGTACTCCGGGACAGAAGTTGGACATAATAGGCACAGACCTCCCTGATGATTCACCCGTCTTAGTGTACATCCACGGCGGCTATTGGCAGATGCTGTCGCGGGAAGCATCGCGGTACCTCGCCCAGCCGCTGCACCGGTTCCGCATCAAGACCATTGTAGTGGGCTACGACCTTTGTCCAGTTGCCACGCTGCCGGAAATAGTCAACCAAATACAGAACGCTGCCAAATTCGTATTCGAGTACGCAGAGAAGATGGGCTCGAGAGGCGTCTACTTCGCCGGCCACTCAGCGGGAGCTCATCTAGTAGCAAAGTTGCTGTCCAATTCAGATTTCCTCGACAGCACAGAAGGTTCCCACCGTCTGCAAGGTGCCTTCCTGATCTCAGGAATCTTCGACTTAAGAGAAGTCATCCATACATCTGTGAACCTTGCAGTGCAGTTGCCCGTCGAATGGGCAGTGCCTCTATCTCCTCAGTTTGACTGCTTCACGCATCTGCAAGCAAGACGAGTGCGTGTATACATTTTAGCGGGACAGTATGATAGCCCGACGTTCAAGAAGCAATCGAGAGAGTTCTACGAACTCTTGCACAATACCTGTTTGATGCAAAACATGTACTTGGAAATCAAAGATAATTTTGACCATTTTGACATCGTGGAGTGTTTTGCCCAAGATGACAATTACCTTAGGAAATTATTGGTGCACGATATTCGTAAGCATCTTTAA
